# QSAR-Guided Design of Serotonin Transporter Inhibitors Supported by Molecular Docking and Biased Molecular Dynamics

**DOI:** 10.3390/ph19030444

**Published:** 2026-03-10

**Authors:** Aleksandar M. Veselinović, Giulia Culletta, Jelena V. Živković, Slavica Sunarić, Žarko Mitić, Muhammad Sohaib Roomi, Marco Tutone

**Affiliations:** 1 Department of Chemistry, Faculty of Medicine, University of Niš, 18000 Niš, Serbia; aveselinovic@medfak.ni.ac.rs (A.M.V.); jelena.zivkovic.hemija@medfak.ni.ac.rs (J.V.Ž.); slavica.sunaric@medfak.ni.ac.rs (S.S.); zarko.mitic@medfak.ni.ac.rs (Ž.M.); 2 Dipartimento di Scienze e Tecnologie Biologiche Chimiche e Farmaceutiche (STEBICEF), Università degli Studi di Palermo, via Archirafi, 28, 90123 Palermo, Italy; muhammadsohaib.roomi@unipa.it (M.S.R.); marco.tutone@unipa.it (M.T.)

**Keywords:** serotonin transporter, major depressive disorder, QSAR, molecular modeling, drug design

## Abstract

**Background/Objectives**: Serotonin transporter (SERT) inhibition represents a central pharmacological strategy in the treatment of major depressive disorder. In this study, an integrated computational framework combining quantitative structure–activity relationship (QSAR) modeling, molecular docking analysis, and in silico ADMET profiling was applied to identify and prioritize novel candidate structures. **Methods**: Conformation-independent QSAR models were developed using local molecular graph invariants and SMILES-based descriptors optimized through a Monte Carlo learning procedure, while a genetic algorithm–multiple linear regression (GA–MLR) was employed to derive statistically robust predictive models from a large descriptor pool. Model quality, robustness, and external predictivity were rigorously evaluated using multiple statistical validation criteria. In parallel, a field-based contribution analysis was applied to construct a three-dimensional QSAR model, enabling spatial interpretation of structure–activity relationships. Fragment-level contributions associated with activity enhancement or attenuation were subsequently identified and used to design new candidate inhibitor structures. **Results**: The designed compounds were further evaluated by molecular docking, InducedFit Docking and Binding Pose MetaDynamics (BPMD) into the SERT binding site, providing a structure-based assessment consistent with the trends observed in QSAR modeling. In addition, in silico ADMET analysis was performed to assess key pharmacokinetic and safety-related properties relevant to central nervous system drug development. **Conclusions**: The proposed workflow demonstrates the utility of combining data-driven QSAR modeling with structure-based and pharmacokinetic considerations to rationalize and prioritize novel serotonin transporter-focused scaffold optimization, offering a transferable strategy for early-stage antidepressant drug discovery.

## 1. Introduction

Major depressive disorder (MDD) is a severe and highly prevalent psychiatric condition that profoundly impairs cognitive, emotional, and social functioning, substantially reducing quality of life and productivity [1,2,3,4]. In terms of years lived with disability, MDD represents the leading cause worldwide and remains among the major contributors to the global burden of disease as quantified by disability-adjusted life years (DALYs) [5,6]. The clinical significance of MDD is further underscored by its high morbidity and mortality, as affected individuals exhibit a markedly increased risk of suicidal ideation, suicide attempts, and death by suicide [7,8].

The treatment of MDD is intrinsically complex, typically requiring a combination of psychotherapy and long-term pharmacological intervention [9,10]. A breakthrough in antidepressant therapy occurred with the introduction of fluoxetine in 1988, the first selective serotonin reuptake inhibitor (SSRI), which established serotonin transporter (SERT) inhibition as a central therapeutic strategy [11]. The pharmacological rationale for this approach is grounded in SERT’s pivotal role in regulating serotonergic neurotransmission by clearing serotonin from the synaptic cleft, thereby terminating signal transmission.

Earlier antidepressant classes, such as monoamine oxidase inhibitors (MAOIs), exerted their effects by reducing intraneuronal serotonin degradation via inhibition of monoamine oxidase enzymes. Subsequent generations of antidepressants, including tricyclic antidepressants (TCAs) and SSRIs, act predominantly by increasing synaptic serotonin concentrations through blockade of its reuptake [12,13]. Despite notable improvements in safety and tolerability relative to older agents, currently prescribed antidepressants are still associated with a range of adverse effects, including weight gain, sleep disturbances, gastrointestinal symptoms, and sexual dysfunction, which frequently compromise treatment adherence and clinical outcomes [14,15,16,17]. Consequently, there remains a substantial unmet medical need for the development of novel antidepressant agents with improved efficacy and safety profiles.

Drug discovery and development are inherently time-consuming and resource-intensive processes, constrained by high attrition rates and the need for extensive experimental validation. In this context, chemoinformatics has emerged as an essential component of modern drug discovery, providing in silico methodologies that enable the rational exploration, optimization, and prioritization of bioactive small molecules [18,19]. Chemoinformatic approaches are widely applied both to the identification of novel lead compounds and to the optimization of pharmacological or pharmacokinetic properties within chemical series exhibiting established biological activity.

Among these approaches, molecular docking, molecular dynamics simulations, and quantitative structure–activity relationship (QSAR) modeling are among the most widely used and conceptually complementary techniques. Docking, and molecular dynamics methods offer structure-based insights into ligand–target interactions [20,21,22,23,24], while QSAR models provide data-driven relationships between chemical structure and biological activity. In contemporary QSAR studies, predictive models are constructed using diverse classes of molecular descriptors derived from well-defined molecular representations, each capturing specific structural, physicochemical, or topological features of the compounds under investigation. These descriptors are subsequently integrated into mathematical models that quantitatively relate molecular characteristics to biological activity, enabling interpretation, prediction, and rational design within a defined chemical domain [25,26,27,28,29].

In the present study, an integrated in silico strategy was employed to explore and characterize small molecules with prospective serotonin transporter inhibitory activity. The QSAR modeling framework was designed to capture structure–activity relationships at multiple levels of molecular representation, combining conformation-independent descriptors derived from SMILES notation and local molecular graph invariants with data-driven optimization, as well as complementary two-dimensional and three-dimensional structure-based perspectives. This multi-level approach was adopted to balance predictive performance with mechanistic interpretability and to reduce method-specific bias.

The central aim of the study was to identify molecular fragments and structural features that govern serotonin transporter inhibition and to examine the degree of concordance among different computational viewpoints. By extracting fragment-level contributions associated with activity enhancement or attenuation, the analysis sought to define interpretable design rules that extend beyond purely statistical correlations. These insights were subsequently used to guide the computer-aided design of new candidate inhibitor compounds with optimized structural characteristics. To support the QSAR-derived findings, molecular docking, InducedFit docking and Binding Pose MetaDynamics (BPMD) were applied as a complementary structure-based analysis, enabling evaluation of the binding plausibility of the designed compounds within the serotonin transporter binding site. Rather than serving as an isolated validation step, docking studies and BPMD simulations were used to assess the qualitative consistency between predicted binding modes and the structure–activity trends identified by QSAR modeling, thereby contributing to a coherent and integrated interpretation of the computational results.

## 2. Results and Discussion

**Figure 1:**
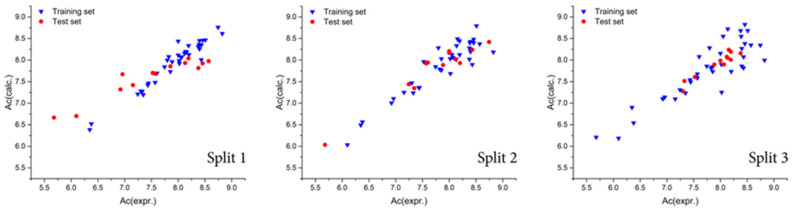
Graphic representation of the best Monte Carlo optimization runs (highest r^2^ values) for the developed conformation-independent QSAR models.

The statistical performance of the conformation-independent QSAR models developed using the Monte Carlo optimization approach is summarized in Table 1. Evaluation of the applied validation metrics demonstrates that the generated models exhibit both high predictive capability and satisfactory reproducibility across all data splits. Among the three examined splits, the most statistically robust model was obtained for the second split, corresponding to a threshold value (T) of 1 and an optimization epoch number (Nepoch) of 10. This parameter combination provided the most favorable balance between model fitting and external predictive performance, as reflected by the statistical criteria employed. This conclusion is supported by a quantitative comparison of the external validation metrics across the three data splits (Table 1). On average, split 2 shows superior predictive performance, with higher test-set values of r^2^ = 0.8992, q^2^ = 0.8725, CCC = 0.9137, and IIC = 0.9479, compared to split 1 (r^2^ = 0.8799, q^2^ = 0.8195, CCC = 0.8016, IIC = 0.9380) and split 3 (r^2^ = 0.8835, q^2^ = 0.8355, CCC = 0.9269, IIC = 0.9396). In addition, split 2 shows a lower mean absolute error on the external test set (MAE = 0.235) than split 1 (MAE = 0.372), while maintaining a balanced relationship between training and test-set statistics. Collectively, these metrics indicate that split 2 offers the best compromise among predictive accuracy, robustness, and generalization ability across the examined data partitions. Applicability domain (AD) analysis indicated that all compounds included in the dataset fell within the defined domain of the developed models, and no outliers were identified. This finding confirms that the chemical space covered by the dataset is coherently represented by the descriptor framework used in the Monte Carlo QSAR modeling, supporting the reliability of the predictions within this domain. A graphical representation of the best-performing QSAR model, corresponding to the highest coefficient of determination (r^2^) achieved in the optimal Monte Carlo optimization run, is presented in Figure 1 for all three dataset splits. The close agreement between observed and predicted pIC_50_ values across both training and test sets visually supports the statistical robustness of the developed models. Model reproducibility was further assessed using the concordance correlation coefficient (CCC), which provides a stringent measure of agreement between predicted and experimental values. The high CCC values across all models indicate strong reproducibility and consistency, reinforcing the reliability of the Monte Carlo-based QSAR approach. In addition, evaluation using MAE-based criteria classified the models as GOOD, providing further evidence of their predictive accuracy and practical utility. To rigorously assess the possibility of chance correlation and overfitting in Monte Carlo-optimized QSAR models, Y-randomization (response permutation across 1000 trials in 10 independent runs) tests were performed on the best-optimized run across three independent data splits (Table S2). In this procedure, the pIC50 values were randomly permuted while preserving the descriptor matrix, and the modeling protocol was repeated. For all three splits, the original models (Run 0) exhibited high predictive performance (training r^2^ = 0.767−0.935; test r^2^ = 0.890–0.909). In contrast, models built on randomized response data showed very low training r^2^ values (Rr2 ≈ 0.02–0.03), indicating the absence of systematic structure–activity relationships under scrambling conditions. Although occasional higher test r^2^ values were observed in isolated randomized runs, these are attributable to the small test set size and are not accompanied by meaningful training performance. Importantly, the CRp^2^ values (0.756–0.924) substantially exceed the commonly accepted threshold of 0.5, confirming that the predictive performance of the reported models is not due to chance correlation. These results collectively support the statistical robustness and reliability of the Monte Carlo QSAR models. The resulting statistics, reported in Table S2, show a pronounced degradation of model performance upon scrambling, confirming that the original models are not the result of chance correlations and retain genuine structure–activity information. Finally, the predictive quality of the QSAR models was assessed using the index of ideality of correlation (IIC), which integrates correlation strength and prediction error into a single metric. The obtained IIC values further support the conclusion that the developed conformation-independent QSAR models possess high predictive potential and are suitable for subsequent interpretation and application in structure-guided ligand design.

The mathematical representations of the best QSAR models, according to the obtained test set r^2^ for all the splits, are presented in Equations (1)–(3).

Split 1: pIC_50_ = 1.88 (±0.04) + 0.026 × DCW (1, 8) (1)

Split 2: pIC_50_ = 3.73 (±0.03) + 0.039 × DCW (1, 10) (2)

Split 3: pIC_50_ = 0.44 (±0.12) + 0.036 × DCW (1, 13) (3)

The presented Equations (1)–(3) indicate that, in the case of split 1, the preferable values for T and N_epoch_ are 1 and 8, respectively; The preferable values of T and N_epoch_ for split 2 are 1 and 10, respectively; and in the case of split 3, the preferable values for T and N_epoch_ are 1 and 13, respectively. The mathematical equations that represent the developed QSAR models generated via GA-MLR modeling for all the splits are illustrated by Equations (4)–(6), and their graphical representation is provided in the Supplementary Material, while the numerical values for the metrics used for validation are given in Table 2.

Split 1: pIC_50_ = 19.4436 + 6.1825 × AATS6p − 0.108 × AATSC3m − 11.4354 × GATS1s − 0.1166 × VR3_Dzs − 10.2573 × SpMin7_Bhi (4)

Split 2: pIC_50_ = 260.9643 − 0.2356 × AATS5i + 2.7227 × ATSC1c − 12.0615 × SpMax1_Bhe + 11.1456 × minHCsatu − 387.1381 × ETA_Epsilon_3 (5)

Split 3: pIC_50_ = 41.5978 + 8.8771 × AATS5p + 5.6828 × MATS1c − 11.0839 × SpMax1_Bhi + 8.5134 × minHCsatu − 0.0069 × fragC (6)

The numerical values of all calculated statistical parameters reported in Table 2 indicate that the GA–MLR-based QSAR models exhibit satisfactory predictive performance and robustness across all three dataset splits. For each split, the applied internal and external validation metrics confirm that statistically reliable and predictive models were obtained, supporting the suitability of the GA–MLR approach for modeling serotonin transporter inhibition within the investigated chemical space.

For the first split (Equation (10)), model performance was determined by a combination of autocorrelation- and eigenvalue-based descriptors: AATS6p, AATSC3m, GATS1s, VR3_Dzs, and SpMin7_Bhi. The positive coefficient for AATS6p indicates that increased medium-range polarizability-weighted autocorrelation (lag 6) is associated with higher predicted activity. Chemically, this suggests that the spatial distribution of polarizable fragments across the scaffold—particularly within extended aromatic systems—enhances interactions compatible with the SERT binding environment. In contrast, AATSC3m (centered mass-weighted autocorrelation, lag 3) carries a negative coefficient, implying that increased short-range mass distribution effects are associated with reduced potency. This may reflect the unfavorable impact of locally concentrated heavy substituents that perturb optimal molecular balance. The descriptor GATS1s, a Geary autocorrelation term weighted by intrinsic state, also contributes negatively, suggesting that excessive short-range electronic-state disparity may reduce activity. Similarly, VR3_Dzs, a logarithmic Randić-like eigenvector-based index derived from the Barysz matrix and weighted by intrinsic state, encodes global topological–electronic organization; its negative coefficient suggests that increased topological complexity or electronic irregularity is not beneficial within this scaffold framework. Finally, SpMin7_Bhi, representing the smallest absolute eigenvalue of the Burden modified matrix weighted by relative first ionization potential, reflects ionization-related electronic extremity. Its negative contribution indicates that increased ionization-driven electronic asymmetry correlates with reduced predicted potency. Collectively, the descriptor pattern for Split 1 highlights a balance between favorable medium-range polarizability distribution and the avoidance of excessive local mass clustering, electronic disparity, or ionization-driven extremity. These findings support a SAR model in which optimal activity arises from electronically coherent and moderately polarizable scaffold organization rather than from highly uneven electronic or topological profiles.

In the second split (Equation (11)), which showed the best statistical performance among the evaluated models, the QSAR equation incorporated AATS5i, ATSC1c, SpMax1_Bhe, minHCsatu, and ETA_Epsilon_3. Importantly, the signs of the regression coefficients allow a more chemically meaningful interpretation. The descriptor AATS5i (average Broto–Moreau autocorrelation lag 5, weighted by ionization potential) has a negative coefficient, indicating that greater long-range, ionization-potential-weighted electronic correlation is associated with reduced predicted potency. Within this scaffold series, excessive propagation of ionization-related electronic effects across the aromatic framework may disrupt the optimal electronic balance required for favorable receptor interaction. In contrast, ATSC1c (centered autocorrelation lag 1 weighted by atomic charges) contributes positively, suggesting that short-range charge distribution plays a beneficial role. This implies that locally organized charge environments—likely influenced by substituent positioning and heteroatom presence—enhance predicted activity within the defined chemical space. The descriptor SpMax1_Bhe, representing the largest absolute eigenvalue of the Burden matrix weighted by Sanderson electronegativities, carries a negative coefficient. This indicates that increased electronegativity-driven spectral extremity correlates with reduced activity, supporting the notion that overly pronounced electronegative topological patterns are unfavorable in the predominantly hydrophobic SERT binding environment. The positive contribution of minHCsatu (minimum E-State value for hydrogen atoms on sp^3^ carbons bonded to unsaturated carbons) suggests that specific aliphatic–unsaturated junction environments are beneficial. In the present scaffold, such structural motifs may contribute to favorable conformational flexibility or appropriate hydrophobic–electronic balance. Finally, ETA_Epsilon_3, an electronegativity-related ETA-family descriptor, exhibits a strongly negative coefficient. This indicates that increases in this topochemical/electronic measure are associated with diminished potency, reinforcing the observation that excessive global electronegativity or topochemical imbalance is detrimental in this series. Overall, the descriptor pattern in Split 2 reflects a nuanced balance between favorable localized charge organization and controlled global electronic distribution. The superior predictive performance of this split likely arises from its ability to capture both short-range charge effects and longer-range electronic/topological constraints in a chemically coherent manner.

For the third split (Equation (12)), the developed model incorporated AATS5p, MATS1c, SpMax1_Bhi, minHCsatu, and fragC. As in previous splits, interpreting the regression coefficients provides insight into the structure–activity trends captured by the model. The descriptor AATS5p (average Broto–Moreau autocorrelation lag 5, weighted by polarizabilities) carries a positive coefficient, indicating that an enhanced distribution of medium-range polarizability across the molecular framework favors increased predicted potency. In the context of the present aromatic-rich scaffold, this suggests that appropriately distributed polarizable substituents contribute beneficially to receptor interaction, potentially through improved dispersion-driven complementarity within the binding pocket. Similarly, MATS1c (Moran autocorrelation lag 1, weighted by atomic charges) shows a positive contribution, underscoring the importance of short-range charge organization. This aligns with the notion that localized charge environments and substituent-induced electronic modulation near key structural junctions are relevant determinants of activity in this series. In contrast, SpMax1_Bhi, the largest absolute eigenvalue of the Burden modified matrix weighted by relative first ionization potential, enters the model with a negative coefficient. This indicates that increased ionization-driven spectral extremity correlates with reduced predicted potency, reinforcing the pattern observed in other splits that excessive global electronic imbalance is detrimental within this scaffold framework. The descriptor minHCsatu, also present in Split 2, contributes positively, supporting the idea that specific sp^3^–unsaturated junction environments are favorable for activity. These motifs may influence conformational adaptability and the hydrophobic–electronic balance required for optimal ligand accommodation. Finally, fragC, a fragment-based complexity descriptor, exhibits a small negative coefficient, suggesting that increased structural fragmentation or excessive molecular complexity provides only marginal or unfavorable contribution to activity within this controlled chemical space. Overall, the descriptor profile of Split 3 underscores the importance of a balanced polarizability distribution and a localized charge organization, while avoiding excessive global electronic extremity or unnecessary structural complexity. Although the predictive stability of this split was slightly lower than that observed for Split 2, the chemical trends remain consistent across models, further supporting the robustness of the derived SAR interpretation.

A comparative analysis of statistical metrics across modeling strategies shows that the second split consistently yields the most robust results for both conformation-independent Monte Carlo QSAR models and GA–MLR models. This convergence across distinct modeling paradigms supports the stability and generality of the identified structure–activity relationships and indicates that the selected training–test partition offers the most representative coverage of the underlying chemical space.

The applicability domain (AD) analysis further corroborates the reliability of the GA–MLR models. Graphical representations of the AD for all three splits, presented in Figures S1–S3 in the Supplementary Material, confirm that all compounds from the test set fall within the defined domain boundaries, indicating that the descriptor space employed in GA–MLR modeling adequately captures the structural diversity of the dataset and supports the validity of the reported predictions. A few compounds from the training set exceeded the leverage threshold but remained within acceptable residual limits, indicating structural influence without response deviation.

The developed three-dimensional QSAR model demonstrated good predictive performance within the studied chemical series. In the training set, the model achieved a high correlation coefficient (r^2^ = 0.9040) with a low standard deviation (SD = 0.2228), indicating a strong descriptive fit to the observed activity values. Importantly, predictive performance remained satisfactory in the independent test set (r^2^ = 0.8456; SD = 0.2824), suggesting that the model retains generalization capability and that its performance is not solely driven by fitting noise in the training data. Model development and selection were conducted within Schrödinger Maestro using the OPLS3 force field and PLS regression over Gaussian field descriptors. To reduce the risk of overfitting, the final model was not selected exclusively on the basis of maximizing r^2^. Instead, the number of PLS factors was determined by balancing goodness-of-fit, cross-validated predictivity (R^2^-CV/q^2^), stability metrics, and scrambling statistics. This multi-criterion selection strategy is particularly important in 3D-QSAR modeling, where increasing the number of latent factors can artificially inflate r^2^ without improving—and sometimes even degrading—predictive robustness. Overall, the close agreement between training and test set statistics, together with the applied cross-validation and stability-guided factor selection, supports the reliability of the derived 3D-QSAR trends within the defined applicability domain. Nevertheless, given the moderate dataset size, the model should be interpreted as a robust series-specific prioritization and SAR rationalization tool rather than as a universally transferable predictor across structurally unrelated SERT inhibitor classes.

Analysis of Gaussian field fraction contributions provides further mechanistic insight into the dominant interaction patterns governing serotonin transporter inhibition. Among the evaluated interaction fields, steric contributions were the most influential, accounting for 47.61% of the total model variance. This dominant steric component underscores the critical role of molecular size, shape, and spatial complementarity in ligand recognition within the serotonin transporter binding pocket. Hydrophobic interactions accounted for the second-largest contribution (22.01%), underscoring the importance of nonpolar contacts and lipophilic surface complementarity in stabilizing ligand–transporter complexes.

In contrast, electrostatic interactions accounted for a relatively minor fraction of the model variance (9.49%), while hydrogen bond acceptor (14.90%) and hydrogen bond donor (5.98%) fields played secondary roles. The comparatively low contribution of electrostatic and hydrogen-bond donor interactions suggests that polar, highly directional interactions are less critical determinants of activity in the studied ligand series. Instead, the results point toward a binding mode primarily driven by steric accommodation and hydrophobic packing, with hydrogen bonding acting as a fine-tuning rather than a governing factor.

**Figure 2:**
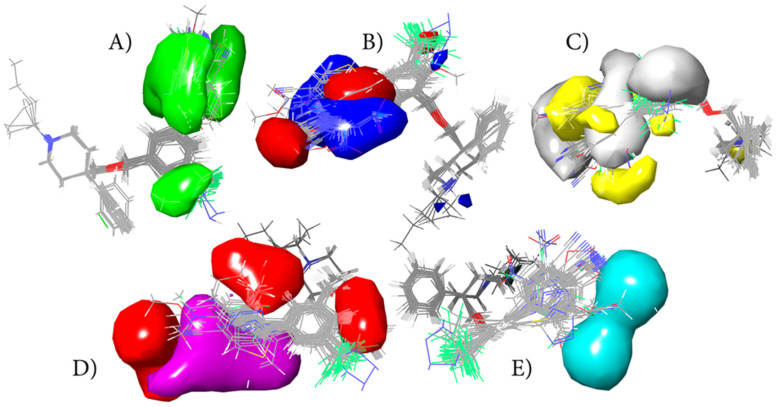
3D QSAR model fields (fields are shown as surfaces). (**A**) Steric—favorable regions (green); (**B**) Electrostatic—favored electropositive (blue) and disfavored electronegative (red); (**C**) Hydrophobic—favored (yellow) and disfavored (white); (**D**) Hydrogen bond acceptor—favored (red) and disfavored (magenta); (**E**) Hydrogen bond donor—disfavored (cyan).

From a design perspective, these findings imply that activity enhancement within this chemical series is more effectively achieved by modulating substituent bulkiness and hydrophobic character than by introducing strongly polar or hydrogen-bond-donating functionalities. This interpretation is fully consistent with the fragment-level trends identified in the conformation-independent and GA–MLR QSAR analyses, as well as with the docking-derived interaction patterns. The three-dimensional contour maps of the steric, electrostatic, hydrophobic, and hydrogen-bonding fields for the optimized 3D QSAR model are shown in Figure 2. These surfaces visually delineate regions where specific interaction types are favorable or unfavorable for activity and provide spatial guidance for further rational optimization of serotonin transporter inhibitors.

One of the central objectives of this study was to identify molecular fragments, expressed as optimal SMILES-based descriptors, that exert either a positive or a negative influence on serotonin transporter inhibitory activity [33,34,35]. In this context, the conformation-independent QSAR framework enables direct association of individual SMILES features with activity-enhancing or activity-attenuating effects, thereby providing mechanistically interpretable insight at the fragment level. The complete set of calculated molecular descriptors, including both SMILES-based descriptors and molecular graph-based local invariants, is reported in Table S3 (Supplementary Material). While graph-based descriptors contribute to the overall predictive performance of the models, SMILES-derived descriptors offer a more intuitive interpretation because they correspond directly to chemically meaningful fragments.

To illustrate the practical interpretation of the Monte Carlo-derived optimal descriptors, a representative example of the calculation of the summarized descriptor correlation weight (DCW) in relation to the biological activity (pIC_50_) is provided in Table 3. For clarity and ease of interpretation, only SMILES-based descriptors were considered in this example, whereas molecular graph-based descriptors were omitted. This presentation highlights how individual fragment contributions are aggregated within the DCW framework and demonstrates the direct link between specific structural motifs and the observed inhibitory activity.

Fragment-level interpretation of the QSAR models enabled identification of SMILES-defined molecular motifs associated with either favorable or unfavorable contributions to inhibitory activity. SMILES fragments corresponding to aliphatic carbon environments, such as “C............” and “C...C.......”, which chemically translate to methyl and ethyl substituents, were consistently associated with positive contributions to pIC_50_. These fragments reflect the beneficial effect of small alkyl groups, which increase local steric bulk and hydrophobic surface area without introducing excessive polarity. Such modifications are consistent with the steric-dominant interaction pattern revealed by the 3D QSAR model. A particularly important class of activity-enhancing fragments consisted of aromatic SMILES motifs, including “c...1...(...”, “c...(...1...”, “c...(...C...”, “c...C...(...”, and “c...1...C...”. These fragments correspond to substituted benzene rings bearing alkyl substituents, most commonly methyl groups, which induce ring branching. From a chemical perspective, these modifications increase aromatic substitution density and steric contouring, thereby improving shape complementarity with the hydrophobic regions of the serotonin transporter binding pocket. This interpretation is in excellent agreement with the strong steric (47.6%) and hydrophobic (22.0%) field contributions observed in the 3D QSAR model. Additional SMILES fragments such as “(...(.......”, “(...........”, and “(...C...(...”, which are associated with molecular branching, further support the conclusion that increased three-dimensionality and steric complexity favor enhanced inhibitory activity. These branching motifs likely promote improved packing within the transporter cavity and restrict unfavorable ligand conformations.

Halogen substitution emerged as another favorable design element. SMILES fragments such as “F...........”, “c...F.......”, and “F...c...1...” correspond to the introduction of fluorine atoms on aromatic rings. Fluorination is known to modulate lipophilicity, electronic distribution, and metabolic stability, and in this series it consistently exerted a positive effect on pIC_50_. Chemically, fluorine substitution may enhance hydrophobic contacts and subtly alter π–π and σ–π interactions within the binding site, while maintaining minimal steric penalty.

**Figure 3:**
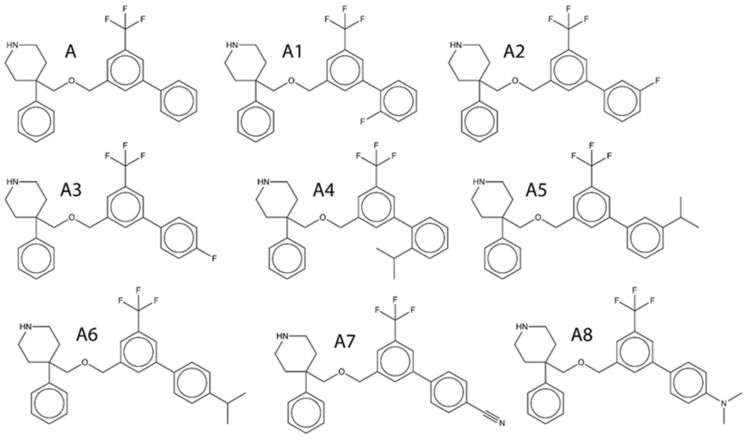
Chemical structures of the designed molecules.

These favorable fragments were systematically introduced at different positions and in varying numbers onto the template molecule **A**, which was selected for structural modification due to its sufficient activity and structural flexibility, which allow systematic optimization. This design strategy yielded eight new candidate molecules (**A1**–**A8**). The chemical structures of the designed compounds are shown in Figure 3, while their calculated pIC_50_ values are summarized in Table 4.

With the exception of molecule **A5**, all designed compounds exhibited higher calculated pIC_50_ values relative to the parent scaffold A, indicating improved inhibitory activity. Molecules **A1**–**A6** incorporated either fluorine or isopropyl substituents at ortho, meta, or para positions on the aromatic ring. An interesting and consistent positional effect was observed, with substitution at the meta position proving the least favorable. In the case of molecule **A5**, meta substitution resulted in a calculated pIC_50_ lower than that of the parent molecule A, highlighting the sensitivity of activity to substitution topology. Comparison of molecules **A1**–**A3** further illustrates this effect. Although these compounds differ only in the positional arrangement of the fluorine atom on the benzene ring, as reflected in the SMILES fragments “Fc1ccccc1c1”, “Fc1cccc(c1)c1”, and “Fc1ccc(cc1)c1”, substantial differences in calculated activity were observed. This behavior arises from the fact that numerous descriptors—both favorable and unfavorable—are generated from these positional SMILES variants, making it impractical to attribute the reduced activity of meta substitution to a single descriptor. Instead, the observed trend reflects a cumulative, context-dependent descriptor effect, underscoring the value of holistic QSAR interpretation over single-fragment reasoning. Beyond steric and hydrophobic optimization, polar functionalization was explored by introducing nitrogen-containing groups. Molecule **A7** incorporated cyano-related SMILES fragments (“N...#.......”, “N...#...C...”), which were associated with positive contributions to pIC_50_ and resulted in higher predicted activity relative to molecule A. The cyano group acts as a strong hydrogen bond acceptor and introduces localized polarity without significantly increasing steric bulk, thereby complementing the predominantly hydrophobic binding environment. Similarly, molecule **A8** introduced substituted amine fragments (“N...........”, “N...(.......”, “N...C.......”), which also exhibited positive contributions to activity. These fragments introduce hydrogen bond acceptor functionality and may participate in secondary polar interactions within the transporter binding site, providing an alternative route to activity enhancement distinct from purely steric modulation. Overall, the CAD results are fully consistent with the conclusions drawn from the 3D QSAR analysis. Molecules **A4**–**A6** primarily exploit steric and hydrophobic optimization, in line with the dominant steric field contribution, whereas molecules **A7** and **A8** introduce hydrogen bond acceptor functionalities that align with secondary polar interaction fields. This convergence between fragment-based QSAR interpretation, three-dimensional field analysis, and rational molecular design underscores the internal consistency of the applied computational strategy and supports the validity of the proposed structure–activity relationships. 

To complement the QSAR-guided computer-aided design and to provide a structure-based perspective on the observed structure–activity trends, molecular docking analysis was employed as a qualitative and comparative tool. Docking was used to explore plausible binding modes of the designed serotonin transporter inhibitors and to examine whether the relative docking score functions and interaction patterns were consistent with the QSAR-predicted pIC_50_ ranking. Importantly, docking was not used as a quantitative predictor of biological activity, but rather as supportive evidence to facilitate the structural interpretation of QSAR-derived activity trends within the serotonin transporter binding site. The calculated docking scores and individual interaction components are summarized in Table 5, with the corresponding predicted inhibitory activities (pIC_50_). 

To examine the relationship between structure-based binding estimates and ligand-based activity predictions, correlation analyses were performed between docking scores (MolDock and Rerank) and the calculated pIC50 values for compounds **A**–**A8**. A statistically significant inverse correlation was observed between the MolDock score and predicted potency (Pearson r = −0.689, *p* = 0.040; Spearman ρ = −0.717, *p* = 0.030), indicating that more favorable (more negative) docking scores tend to be associated with higher predicted inhibitory activity within the series. A comparable trend was obtained for the Rerank score (Pearson r = −0.699, *p* = 0.036), with an even stronger monotonic relationship observed in the rank-based analysis (Spearman ρ = −0.817, *p* = 0.007). Scatter plots illustrating these relationships are provided in the Supplementary Information, Figure S4. Although docking scoring functions are not intended to yield quantitatively accurate binding affinities, the observed moderate-to-strong inverse correlations suggest internal consistency between the structure-based docking results and the QSAR-derived potency predictions. In particular, the stronger rank-based correlation observed for the Rerank score may reflect improved sensitivity to steric and interaction-related contributions within the binding pocket. It is important to note that the dataset comprises a limited number of compounds (n = 9), which restricts statistical power and precludes overinterpretation of correlation strength. Accordingly, docking was primarily used for structural interpretation and binding-mode rationalization rather than as an independent predictive model. Nevertheless, the statistically significant trends observed across both scoring functions support the mechanistic plausibility of the QSAR-derived activity estimates and reinforce the coherence of the proposed structure–activity relationships within the designed compound series.

**Figure 4:**
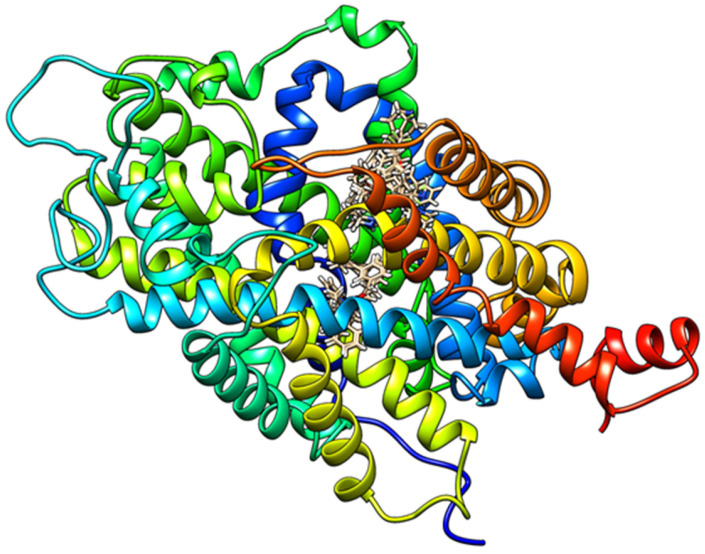
The best-calculated poses for all the designed molecules within the active site of the serotonin transporter.

Overall, the docking results exhibit a consistent qualitative agreement with the QSAR-derived activity trends. Compounds with higher calculated pIC_50_ values generally exhibit more favorable composite docking scores, particularly for steric, van der Waals, and Rerank contributions, supporting the notion that improved inhibitory activity in this series is primarily driven by enhanced shape complementarity and hydrophobic packing within the serotonin transporter binding site. Among the designed molecules, compounds **A3**, **A6**, **A7**, and **A8** stand out as top-performing candidates in both QSAR prediction and docking evaluation. Molecule **A3**, which shows a high predicted activity (pIC_50_ = 8.1855), exhibits a markedly favorable steric contribution (−203.228 kcal/mol) and a strongly negative van der Waals term (−43.7415 kcal/mol), resulting in one of the most favorable MolDock (−174.068 kcal/mol) and Rerank (−139.04 kcal/mol) scores within the series. These results indicate efficient occupation of the binding cavity and optimal steric accommodation, consistent with the dominant steric field contribution identified in the 3D QSAR analysis. A similar pattern is observed for molecule **A6** (pIC_50_ = 8.0295 kcal/mol), which combines a highly favorable steric term (−189.35 kcal/mol) with one of the most negative van der Waals contributions (−50.3446 kcal/mol), leading to a low Rerank score (−138.993 kcal/mol). The absence of significant hydrogen bond contributions for this compound further supports a binding mode dominated by hydrophobic and steric interactions, in full agreement with the QSAR-based interpretation. Molecules **A7** and **A8**, which display the highest predicted activities in the series (pIC_50_ = 8.1943 and 8.4814, respectively), show slightly different but complementary docking signatures. While their steric contributions remain strongly favorable (−182.255 kcal/mol for **A7** and −194.602 kcal/mol for **A8**), these compounds additionally benefit from polar interaction terms. In particular, the presence of cyano (**A7**) and tertiary amine (**A8**) functionalities introduces hydrogen-bond acceptor character, reflected in nonzero hydrogen-bonding and NoHBond contributions. This mixed interaction profile suggests that, although steric and hydrophobic effects remain dominant, secondary polar interactions may further stabilize ligand binding and contribute to the superior predicted activity of these compounds. In contrast, molecule **A5** represents an instructive negative example. Despite exhibiting reasonable steric contributions (−181.313 kcal/mol), **A5** shows a substantially less favorable van der Waals term (25.7723 kcal/mol) and the least favorable Rerank score (−89.5642 kcal/mol) among the designed compounds. This unfavorable interaction pattern is consistent with its lower predicted activity (pIC_50_ = 7.6785) relative to the parent molecule **A** and underscores the detrimental effect of meta substitution observed in the QSAR-guided design phase. The docking results thus reinforce the conclusion that improper substituent positioning can disrupt optimal packing within the binding site, even when overall molecular size and composition are comparable. The parent molecule **A** and the moderately active derivatives **A1** and **A2** occupy an intermediate position in both docking scores and predicted activity. Although these compounds exhibit acceptable steric interactions, their overall MolDock and Rerank scores are lower than those of the top-performing analogs, reflecting suboptimal engagement of the transporter’s hydrophobic binding pocket. Taken together, the docking analysis supports the QSAR- and CAD-derived structure–activity relationships by demonstrating that increased predicted inhibitory activity correlates with improved steric and hydrophobic complementarity within the serotonin transporter binding site. The convergence of docking scores with calculated pIC_50_ values strengthens confidence in the designed compounds and highlights steric optimization, complemented by strategically placed polar functionalities, as an effective strategy for enhancing serotonin transporter inhibition. All the interactions between the selected molecules and the amino acids from the active site of the serotonin transporter have been identified. Moreover, two-dimensional representations of hydrogen bonding, hydrophobic, and hydrophilic interactions between the ligands and the serotonin transporter binding site are provided in the Supplementary Information, Figures S5–S13, while the best-ranked docking poses of all designed molecules within the active site are shown in Figure 4. Analysis of docking poses reveals two distinct binding clusters within the transporter cavity. Molecules **A**, **A2**, and **A4** preferentially occupy one region of the binding site, whereas molecules **A1**, **A3**, **A5**, **A6**, and **A7** are positioned in an alternative subpocket. This spatial segregation indicates that structurally related compounds may adopt different binding orientations while maintaining comparable interaction patterns dominated by steric and hydrophobic complementarity. Importantly, the existence of multiple binding clusters supports the QSAR- and 3D QSAR-derived conclusions that activity within this chemical series is not governed by a single, highly specific interaction motif, but rather by overall shape accommodation and hydrophobic packing within the serotonin transporter binding cavity. The observed clustering behavior further suggests a degree of binding site plasticity, which may accommodate structurally diverse ligands through alternative, yet energetically favorable, binding modes. 

In traditional molecular docking studies, we observe how a ligand binds in a pocket. However, docking results cannot be validated solely by the ligand’s binding to the receptor in a specific binding pocket. In silico analysis has shifted its focus from simple docking poses to binding time. A drug that stays bound to its target for a longer period often has better efficacy in the body. To understand the stability and effectiveness of the complex we use InducedFit Docking and BPMD, a “biased” simulation approach that works by applying an external computational force, a biasing potential to the ligand to encourage it to move out or “un-bind” from its docked position. The results of this study demonstrate how strongly a ligand binds to its receptor and how much energy is required to uncouple the binding of receptor-ligand complex; a higher energy amount required for unbinding indicates a more stable and robust ligand-receptor complex.

In this study, we employed BPMD on the receptor-ligand complexes. This approach applies a biasing potential along a specific Collective Variable (CV), defined by the RMSD of the ligand relative to its initial binding pose. The stability of the complex was evaluated using PoseScore, which tracks the average RMSD from the starting position, where a sharp increase indicates ligand dissociation, and Persistence Score (PersScore), which quantifies the stability of hydrogen bonds throughout the trajectory. The simulation systematically corrects the system for staying in its original state, effectively forcing the ligand to explore new conformations by adding this history-dependent potential.

**Figure 5:**
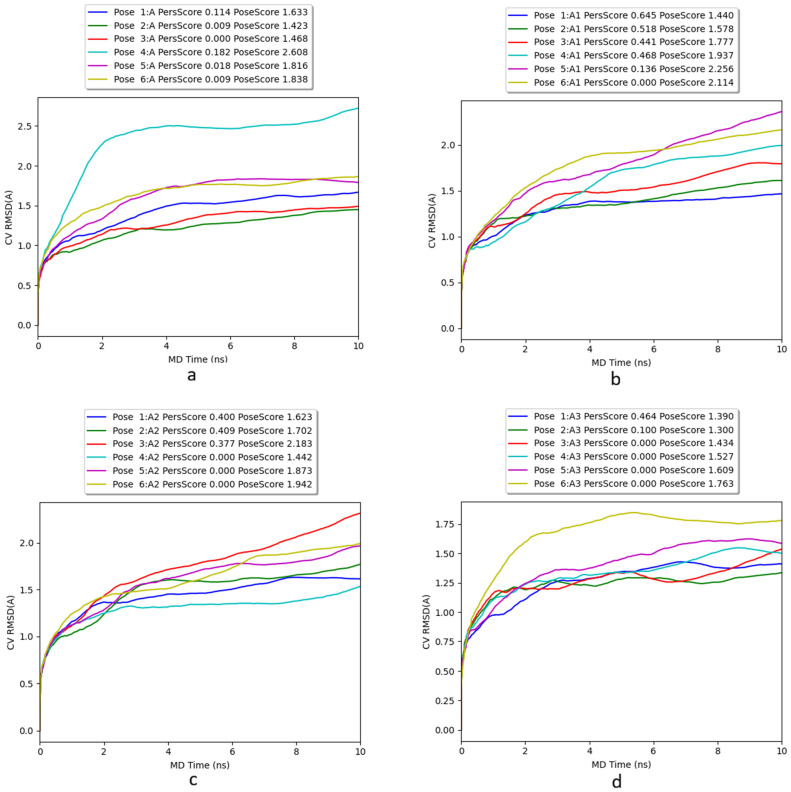

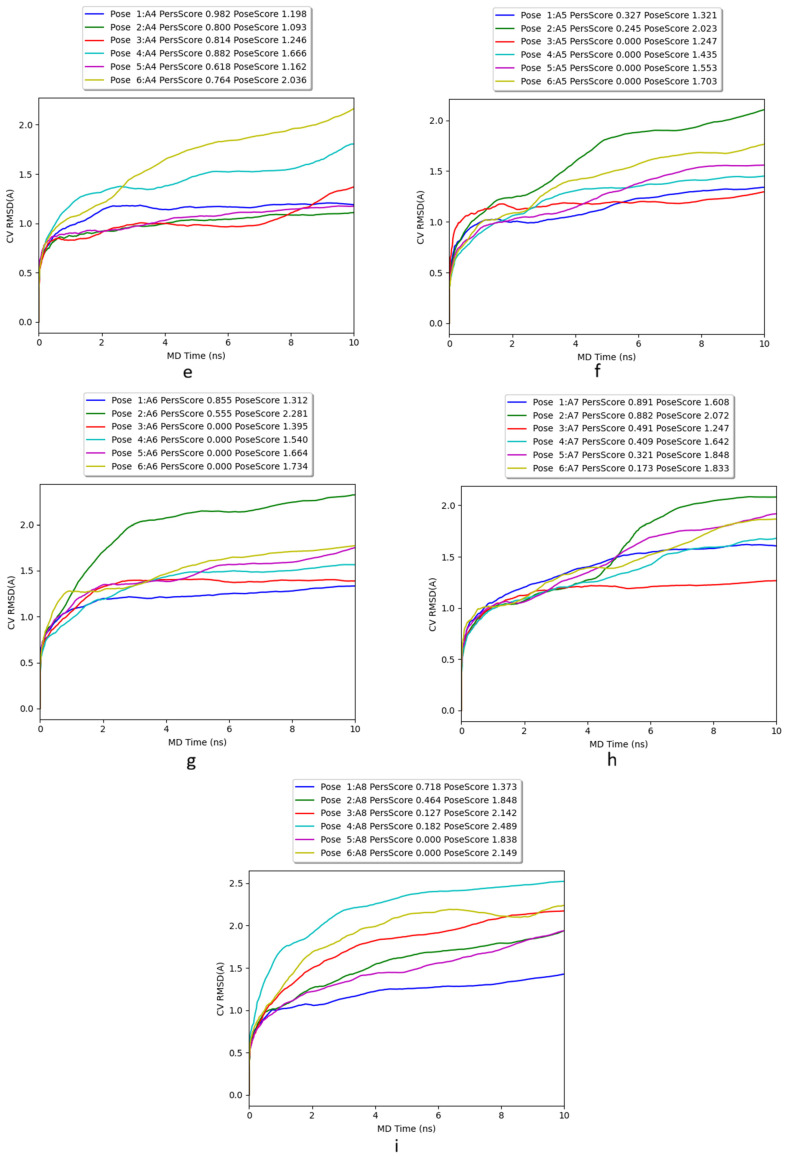
Binding Pose Meta Dynamics (BPMD) analysis of the six best-ranked poses obtained from InducedFit docking of compounds within the receptor (PDB ID: 6AWN): (**a**) compound **A**, (**b**) compound **A1**, (**c**) compound **A2**, (**d**) compound **A3**, (**e**) compound A4, (**f**) compound A5, (**g**) compound A6, (**h**) compound A7, (**i**) compound A8.

In Figure 5, we reported the BPMD plots of the six best-ranked poses obtained from InducedFit Docking. In all the simulations, the six poses for each compound remain into the binding pocket with CV RMSD values (PoseScore) below 2.5 Å. Only one pose for compound **A** and compound **A8** reaches a higher value but is compensated by the other poses that have a PoseScore not higher than 1.75 Å on average. Moreover, CompScore confirms the high binding capability of these compounds as reported in Table 6. The advanced docking results from IFD and BPMD simulations confirm that the compounds identified by QSAR analysis could represent reliable SERT inhibitors.

The in silico ADMET analysis was performed to evaluate the pharmacokinetic suitability and preliminary safety profile of the designed serotonin transporter inhibitors (**A**–**A8**), with particular emphasis on parameters critical for central nervous system (CNS) drug candidates (Table S4). All investigated compounds (**A**–**A8**) were predicted to be well absorbed in the human intestine (HIA: absorbed), indicating favorable oral absorption potential. Predicted Caco-2 permeability values were narrowly distributed, with logPapp ranging from −5.28 to −5.40, suggesting moderate and relatively uniform intestinal epithelial permeability across the series. The lack of pronounced variability in both HIA and Caco-2 permeability indicates that structural modifications introduced in the designed analogs did not adversely affect intestinal absorption. Importantly, none of the compounds were predicted to be P-glycoprotein substrates, while all were classified as P-gp inhibitors. The absence of P-gp substrate liability suggests reduced intestinal efflux and limited active extrusion at the blood–brain barrier, although the predicted P-gp inhibitory behavior may warrant attention in the context of potential drug–drug interactions.

Predicted blood–brain barrier (BBB) permeability was evaluated using the DeepPK platform, which provides both a quantitative CNS-related score and a machine learning-based categorical BBB penetration prediction. The numerical CNS scores for the designed compounds (**A**–**A8**) were distributed within a relatively narrow range (−1.27 to −1.79), indicating homogeneous predicted CNS distribution tendencies across the series. Importantly, according to the categorical classifier output, all compounds were predicted as “BBB Penetrable (High Confidence)”. The relatively consistent CNS-related scores across the series suggest that the introduced structural modifications did not markedly alter predicted brain exposure characteristics relative to the parent scaffold. The compounds exhibit elevated lipophilicity (LogD at pH 7.4 between 4.74 and 5.23) and high plasma protein binding, features frequently observed among clinically used serotonin reuptake inhibitors. In this context, the predicted BBB profile appears to reflect a balance between favorable lipophilicity and distribution-related parameters rather than structural constraints incompatible with CNS penetration. Overall, the BBB assessment supports the internal consistency of the designed series and does not indicate an intrinsic barrier to CNS exposure within the predictive limits of the applied in silico model.

All compounds exhibited very high predicted plasma protein binding (PPB 88.0–95.6%), resulting in relatively low predicted free fractions in human plasma (fraction unbound ≈ 1.8–2.2%). While high PPB is common among CNS-active small molecules, such extensive binding may further limit the free drug concentration available for brain penetration, thereby compounding the unfavorable BBB profile. Predicted steady-state volumes of distribution (Vdss) were generally high (log Vdss ≈ 4.7–5.4 L/kg), suggesting extensive tissue distribution. However, in the absence of effective BBB penetration, high Vdss alone does not translate into meaningful CNS exposure.

From a metabolic perspective, all compounds were predicted to be non-inhibitors of CYP2D6 and CYP3A4 (High Confidence), which is a favorable characteristic given the clinical relevance of these enzymes in antidepressant metabolism and the well-documented risk of drug–drug interactions associated with their inhibition. All compounds were uniformly predicted to be substrates of CYP3A4 (High Confidence), and six of nine compounds (**A**, **A4**–**A8**) were additionally predicted to be substrates of CYP2D6, indicating susceptibility to metabolic clearance via both pathways. This is consistent with the predicted high clearance values (≈5.4–9.4 mL/min/kg) and uniformly short elimination half-lives (<3 h, High Confidence) across the entire series, suggesting rapid systemic elimination. While such profiles may reduce the risk of long-term accumulation, they may also necessitate frequent dosing regimens or sustained-release formulations to maintain adequate therapeutic exposure. All compounds were additionally predicted to be P-glycoprotein inhibitors (High Confidence, probability 0.987–1.000), while none were predicted to be P-gp substrates, a profile that may influence intestinal absorption and CNS penetration and warrants consideration in the context of drug–drug interactions at the efflux transporter level.

From a safety perspective, all compounds were uniformly predicted to be hERG blockers (High Confidence, probability 0.995–0.999), representing the most significant in silico safety liability identified in this analysis. The associated risk of QT interval prolongation and cardiac arrhythmia is a well-recognized challenge in CNS drug development and underscores the need for experimental hERG patch-clamp validation prior to any further advancement of this series. AMES mutagenicity predictions were uniformly negative (Safe, High Confidence), and the DILI I model classified all compounds as non-hepatotoxic. However, the DILI II model predicted hepatotoxic potential for all compounds (probability 0.771–0.867), introducing a degree of ambiguity regarding hepatic safety that the DILI I result alone does not resolve; this discordance between the two hepatotoxicity models is itself an indication that experimental hepatotoxicity profiling should be included in any future in vitro characterization of the series. Additional toxicity signals of note include uniform predictions of micronucleus induction (High Confidence) and respiratory toxicity (High Confidence) across all compounds, as well as thyroid receptor-mediated activity (NR-TR Toxic) for eight of nine compounds—compound **A7** being the sole exception, predicted as Safe with low confidence. Taken together, these findings indicate that while the series does not raise genotoxicity concerns in the AMES assay, a broader toxicological evaluation encompassing clastogenicity, pulmonary safety, and endocrine activity will be an essential component of any experimental follow-up. As with all findings reported in this section, the in silico nature of these predictions necessitates cautious interpretation, and experimental validation remains the definitive criterion for safety assessment.

Overall, ADMET profiling delineates a well-defined, internally consistent pharmacokinetic and safety landscape for the designed serotonin transporter inhibitors. The series is characterized by uniform intestinal absorption, the absence of clinically relevant CYP2D6 or CYP3A4 inhibition liabilities, and consistently low predicted genotoxic and hepatotoxic risks. These features collectively support the scaffold’s developability from an absorption, metabolism, and early safety perspective. At the same time, the ADMET results highlight specific distribution- and safety-related aspects that warrant targeted optimization. In particular, blood–brain barrier permeability and hERG liability emerge as structure-sensitive properties that may benefit from further fine-tuning, rather than constituting disqualifying limitations at this stage. Importantly, the narrow dispersion of ADMET descriptors across the series suggests that rational modulation of molecular polarity, charge distribution, and substituent patterns can be pursued without compromising the favorable absorption and metabolic interaction profile already established. In this context, the ADMET analysis does not serve as a filtering endpoint but rather as a guiding framework for iterative lead optimization, complementing the QSAR- and docking-based activity assessment and informing subsequent computational and experimental refinement steps.

The predicted inhibitory potency values for the designed compounds range from pIC_50_ = 7.68 to 8.48, corresponding to IC_50_ values of approximately 3.30–20.97 nM at SERT. To contextualize these values within the broader pharmacological landscape of serotonin transporter inhibition, they may be compared with the experimentally determined affinities of clinically approved SSRIs reported in the primary literature. Sertraline and Paroxetine are among the most potent clinically used SERT inhibitors, with reported Ki values of 0.1 nM and 0.13 nM, respectively. Escitalopram and R-fluoxetine exhibit Ki values of 1.1 nM and 1.4 nM, respectively, while the fluoxetine racemate has a reported IC50 of approximately 10 nM at human SERT. The predicted IC50 values for the designed compounds therefore fall within the low nanomolar range characteristic of therapeutic SERT inhibition. Compound **A8**, with a predicted IC50 of 3.30 nM, is situated within the activity range of escitalopram and R-fluoxetine, while compounds **A7** and **A3** (predicted IC50 = 6.39 and 6.52 nM, respectively) are comparably positioned. Seven of the nine designed compounds have predicted IC_50_ values below 13 nM. It must be emphasized that these are QSAR-predicted values derived from validated models (external validation r^2^ = 0.880–0.899), not experimentally measured affinities, and that direct comparison with clinical benchmarks is therefore indicative rather than definitive. Nevertheless, the predicted activity range, combined with the independent support from molecular docking and Binding Pose Metadynamics analyses, positions the designed series as computationally prioritized candidates warranting experimental follow-up. The structural distinction between the investigated scaffold and clinically approved SSRIs—which belong to pharmacologically distinct chemical classes—precludes their incorporation into the QSAR regression framework; however, this does not diminish the pharmacological relevance of the predicted activity values, which fall squarely within the therapeutic range established by approved antidepressants.

In addition to the statistical correlation analysis, structural validation was performed to ensure that the docking-derived trends are mechanistically meaningful. The docking protocol was validated by redocking the co-crystallized ligand from the 6AWN structure, yielding a heavy-atom RMSD of 1.72 Å relative to the crystallographic pose, confirming reliable reproduction of the experimental binding mode. Clustering analysis of the docked poses revealed two recurrent orientation patterns within the same orthosteric binding site. Importantly, these clusters correspond to alternative orientations of flexible peripheral substituents while preserving the key anchoring interactions within the binding pocket. To further assess the stability of these binding modes beyond static scoring, the top-ranked poses were subjected to binding pose meta dynamics (BPMD) simulations. The BPMD trajectories demonstrated sustained occupancy of the binding pocket and limited conformational drift, supporting the interpretation that the observed pose clusters represent alternative but energetically viable binding orientations rather than docking artifacts. Taken together, the redocking validation, pose stability analysis, and statistically significant correlation between docking scores and predicted pIC50 values provide convergent evidence for the structural plausibility of the proposed binding models. The present study is computational in nature. The computational workflow presented here is consistent with established practices in ligand-based drug design, where QSAR-guided prioritization and in silico ADMET profiling constitute independent and publishable contributions to early-stage drug discovery, preceding experimental synthesis and biological evaluation. While the QSAR models are grounded in experimentally determined IC50 values reported in the literature, the newly designed analogs remain theoretical candidates and require experimental validation of their biological activity. Clinically used SSRIs (e.g., paroxetine, sertraline, fluoxetine) exhibit distinct scaffold architectures compared to the series investigated herein; therefore, the present work focuses on internal scaffold optimization rather than cross-class comparative modeling. The proposed compounds should therefore be regarded as prioritized hypotheses for future synthesis and pharmacological evaluation rather than confirmed SERT inhibitors.

## 3. Materials and Methods

A dataset comprising 50 small molecules with experimentally established inhibitory activity against the serotonin transporter was compiled from the literature [36]. All compounds were retrieved from the cited literature, which reported experimentally determined IC_50_ values under comparable assay conditions. To ensure dataset consistency, only compounds from the same study were included. Salts were converted to their parent structures, duplicates were removed, stereochemical information was preserved as reported, and activity values were transformed to pIC_50_ prior to modeling. Although the dataset comprises compounds sharing a common central scaffold, structural diversity is introduced through systematic variation in substituents and peripheral aromatic moieties. To provide a quantitative characterization of the structural diversity within the dataset, pairwise Tanimoto similarities were computed for all 1225 unique compound pairs using SMILES-based structural fingerprints. The resulting similarity matrix is presented in Figure S14 (Supplementary Material). The mean pairwise Tanimoto similarity was 0.700 (SD = 0.158), with values ranging from 0.229 to 1.000. Notably, 41.8% of all compound pairs exhibited Tanimoto similarity below 0.70, and 11.8% of pairs fell below 0.50, confirming the presence of structurally distinct compound clusters within the dataset. Structural analysis identified six substituent subclasses represented in the dataset: heterocyclic derivatives bearing tetrazole or triazole moieties (n = 6), ortho-substituted biaryl analogs (n = 7), meta-substituted biaryl analogs (n = 7), para-substituted biaryl analogs (n = 16), polyfluorinated aryl derivatives (n = 9), and N-alkyl piperidine variants (n = 5). This compositional analysis demonstrates that, although all compounds share a common central scaffold, the dataset encompasses meaningful structural variation across multiple substituent dimensions—sufficient to support robust fragment-level SAR extraction within the defined chemical series. This design ensures exploration of a chemically coherent but non-trivial region of chemical space, where activity modulation arises from defined substituent patterns rather than from scaffold hopping. Such controlled diversity is particularly suitable for Monte Carlo-based QSAR modeling, as it allows reliable extraction of fragment-level contributions without the confounding effects of excessive structural heterogeneity. Therefore, while the overall chemical space is intentionally focused, it remains sufficiently variable to enable meaningful structure–activity relationship analysis within the investigated series. Canonical SMILES representations of all compounds, together with their corresponding pIC_50_ values, are provided in Table S1 (Supplementary Material). To enable model development and external validation, the dataset was randomly divided into training and test subsets using three independent splits. In each split, 38 compounds (75%) were assigned to the training set, while the remaining 12 compounds (25%) constituted the external test set. The distribution of biological activity values across the training and test subsets was examined to ensure comparable coverage of the activity range and approximate normality, following a previously reported procedure [37]. This strategy was adopted to reduce selection bias and assess the robustness of the derived models to data partitioning.

### 3.1. QSAR Modeling Based on the Monte Carlo Optimization Method

Monte Carlo optimization was employed for the development of conformation-independent QSAR models using a hybrid descriptor framework that integrates molecular graph-based and SMILES-based representations. This approach enables simultaneous exploitation of topological information encoded in the molecular graph and fragment-level features captured by SMILES notation, while avoiding dependence on three-dimensional conformations. The molecular graph-based component of the descriptor space was constructed using local graph invariants derived from elementary graph-theoretical concepts, such as paths and walks. These invariants encode neighborhood connectivity and topological complexity and have been extensively described in the literature [38]. In the present study, the graph-based descriptor set included extended connectivity indices of increasing order, valence-shell descriptors reflecting local atomic environments, path-based measures capturing short-range connectivity patterns, and atom-type-specific neighbor counts distinguishing between carbon and heteroatom environments. Together, these descriptors provide a compact yet expressive representation of molecular topology relevant to biological activity. In parallel, SMILES-based descriptors were incorporated to introduce a fragment-oriented and mechanistically interpretable dimension to the QSAR models. Unlike purely topological descriptors, SMILES-derived features are directly associated with specific molecular fragments and substructures. Each SMILES descriptor contributes to a correlation weight reflecting its influence on biological activity, and the overall descriptor value for a given molecule is defined as the sum of the correlation weights of all its constituent SMILES features. This cumulative descriptor, referred to as the descriptor correlation weight (DCW), was computed using Equation (7) and subsequently optimized via the Monte Carlo learning procedure.

DCW (T, Nepoch) = zΣCW (ATOMPAIR) +xΣCW (NOSP) + yΣCW (BOND) + tΣCW (HALO) + rΣCW (HARD) + αΣCW (S_k_) + βΣCW (SS_k_) + γΣCW (SSS_k_) (7)

In the Monte Carlo-based QSAR framework, each molecular feature, derived from SMILES notation or a molecular graph representation, is assigned a numerical coefficient, the correlation weight (CW). The CW quantifies both the direction and the magnitude of an individual molecular attribute’s contribution to the modeled biological activity. During Monte Carlo optimization, CW values are iteratively adjusted to maximize the correlation between the molecular descriptor and the experimental endpoint in the training set. Molecular features occurring less frequently than the predefined threshold value (T) are classified as rare and assigned a CW value of zero, whereas sufficiently frequent features are retained and optimized. Positive CW values indicate structural attributes associated with activity enhancement, while negative CW values correspond to features contributing to activity attenuation. The overall molecular descriptor used for QSAR model construction is the descriptor correlation weight (DCW), which represents the cumulative contribution of all active molecular features in a given compound. In Equation (7), the variables z, x, y, t, r, α, β, and γ are binary indicators assuming values of 1 (included) or 0 (excluded), thereby controlling whether a given class of SMILES-based descriptors participates in model development. This binary scheme enables the Monte Carlo optimization procedure to selectively activate or suppress individual descriptor classes based on their contribution to model performance. The symbol S_k_ denotes a SMILES atom represented by a single SMILES symbol or by two inseparable symbols and forms the basis of local SMILES descriptors. In addition to single-atom descriptors, local structural information is captured through linear combinations of two and three adjacent SMILES atoms, represented by SS_k_ and SSS_k_, respectively. These local SMILES descriptors encode fragment-level patterns that are directly related to ligand substructures and provide mechanistically interpretable contributions to biological activity. Beyond local descriptors, the SMILES-based descriptor set also includes global descriptors reflecting overall molecular features. These global SMILES descriptors characterize properties such as atom pair composition (ATOMPAIR), nitrogen–oxygen–sulfur–phosphorus patterns (NOSP), bond-related features (BOND), halogen-related attributes (HALO), and molecular hardness-related characteristics (HARD) and were defined in accordance with previously reported methodology [33]. The combined use of local and global SMILES descriptors enables simultaneous representation of fragment-specific effects and whole-molecule characteristics within a unified, conformation-independent QSAR framework. In the present study, QSAR model development was based on the combined use of SMILES-derived descriptors (local and global) and molecular graph-based local invariants. The optimal descriptor for each molecule, expressed as the descriptor correlation weight (DCW), was computed from the selected SMILES and graph-based contributions using Equation (8). This integrated descriptor framework served as the basis for subsequent model optimization and statistical evaluation.

DCW (T, N_epoch_) = ΣCW (ATOMPAIR) +ΣCW (NOSP) + ΣCW (BOND) + ΣCW (HALO) + ΣCW (HARD) + ΣCW (S_k_) + ΣCW (SS_k_) + ΣCW (SSS_k_) + ΣCW (EC0_k_) + ΣCW (PT2_k_) + ΣCW (PT3_k_) + ΣCW (VS2_k_) + ΣCW (VS3_k_) + ΣCW (NNC_k_) (8)

In addition to the previously defined local SMILES symbols (S_k_, SS_k_, and SSS_k_), several graph-theoretical descriptors are incorporated into Equation (2) to represent molecular topology at different levels of structural resolution. These include the zero-order Morgan connectivity index (EC0_k_), calculated on the hydrogen-suppressed molecular graph, path-based descriptors of lengths two and three (PT2_k_ and PT3_k_), valence shell descriptors of ranges two and three (VS2_k_ and VS3_k_), and nearest-neighbor counts (NNC_k_), all defined according to established graph-theoretical principles [38]. Together, these descriptors complement the SMILES-based representation by encoding local connectivity, branching, and neighborhood effects that may influence biological activity.

All molecular descriptors used in this study were calculated using the CORAL software package (CORrelation and Logic; http://www.insilico.eu/coral (accessed on 7 May 2025)), which implements Monte Carlo-based optimization of descriptor correlation weights. Once an optimal descriptor set is identified via the Monte Carlo procedure, each descriptor is assigned a numerical coefficient, the correlation weight (CW). The assignment and optimization of these correlation weights constitute the core of the Monte Carlo learning process.

The Monte Carlo optimization operates by generating random numerical values and evaluating their impact on the correlation between molecular descriptors and the biological endpoint. In each Monte Carlo run, correlation weights are initially assigned to descriptors at random based on the molecules’ SMILES representations and the specific endpoint under consideration. By iteratively adjusting these weights, the algorithm seeks to maximize the correlation coefficient between the optimal descriptor and the biological activity for the training set. As a result, the optimization process yields a set of correlation weights that provide the best statistical association between molecular structure and activity. Two key parameters govern the behavior and outcome of the Monte Carlo optimization: the threshold value (T) and the number of epochs (Nepoch). The threshold parameter is used to classify molecular features derived from both SMILES notation and SMILES-based molecular fragments into active and rare categories. Specifically, if a given molecular feature (X) occurs fewer than T times in the training set, it is considered rare and excluded from the model by assigning it a correlation weight, CW (X), of zero. Conversely, features occurring at least T times are classified as active and retained for model construction. This thresholding procedure reduces noise from sparsely represented fragments and improves model generalizability. The Nepoch parameter defines the number of optimization cycles performed during the Monte Carlo process and plays a critical role in balancing model fitting and predictive performance. As the number of epochs increases, the correlation coefficient for the training set typically improves and may reach a maximum with an unlimited number of epochs. However, optimal predictive performance on the external test set is generally achieved at a finite, well-defined number of epochs. Beyond this point, further optimization may lead to overfitting, reflected by a decline in test-set correlation despite continued improvement in training-set statistics. Therefore, the optimal value of Nepoch is selected as the epoch that yields the highest correlation coefficient on the external test set. A similar trade-off is observed for the threshold parameter. Increasing T may reduce descriptor noise but can also lead to the exclusion of informative structural features, thereby decreasing the training-set correlation. Nevertheless, for the external test set, a specific threshold value typically exists that maximizes predictive performance. From a practical modeling perspective, this threshold is preferred because it offers the best balance between model simplicity and predictive accuracy. Accordingly, optimal values of both T and Nepoch were systematically explored and selected to construct statistically reliable QSAR models based on combined SMILES-based and molecular graph-based descriptors [33]. In the present study, the search for the optimal parameter combination was conducted over the ranges T = 1–5 and Nepoch = 0–50.

From an algorithmic standpoint, Monte Carlo simulations are based on iterative procedures that explore the distributions of unknown probabilistic variables. In the context of QSAR modeling, this exploration is guided by a defined target function evaluated on the training set. The optimization process begins by initializing the correlation weights for each SMILES attribute (SA) to values of 1 ± 0.01 × Rnd, where Rnd is a uniformly distributed random number between 0 and 1. To avoid systematic bias, the original ordering of SMILES attributes is replaced by a randomized sequence.

Subsequently, the initial value of the target function is computed, followed by iterative updates to the correlation weights to improve its value. This sequence of operations is repeated for all non-rare attributes throughout the Monte Carlo optimization process [33,34]. Once the optimal set of correlation weights has been determined, linear regression is applied to construct the final QSAR model using the training set, as expressed in Equation (9). The resulting model is selected based on its statistical performance for the external test set, ensuring both robustness and predictive relevance.

pIC_50_ = C_0_ + C_1_ × DCW (T, N_epoch_) (9)

### 3.2. QSAR Modeling Based on the Genetic Algorithm, Coupled with Multiple Linear Regression

Two-dimensional molecular descriptors were calculated using the PaDEL-Descriptor software package 2.21 [39]. Descriptor preprocessing was carried out in accordance with standard QSARINS procedures to reduce redundancy and ensure model stability. Constant and near-constant descriptors, defined as those exhibiting variance lower than 0.01 across the dataset, were excluded from further analysis. Subsequently, pairwise correlation analysis was performed to control multicollinearity, and one descriptor from each pair of highly intercorrelated variables (|r| > 0.90) was removed. This preprocessing strategy ensured a reduced, non-redundant descriptor set suitable for robust GA–MLR model development. Subsequent descriptor selection and QSAR model development were performed using the QSARINS (QSAR-INsubria) 2.2.4 software platform [30,31]. After reducing the descriptor pool, the remaining descriptors were scaled to ensure comparability and numerical stability during model construction. QSAR models were then developed using the same training/test set partitions as those applied in the conformation-independent QSAR modeling, thereby enabling a consistent comparison of modeling strategies [32,40]. Within QSARINS, descriptor selection was performed using a genetic algorithm (GA) coupled with multiple linear regression (MLR) as the fitness evaluation function [41,42]. In this framework, the GA iteratively explores combinations of descriptors, while MLR assesses model quality based on statistical performance. The number of descriptors included in each model was fixed at four, in order to balance model simplicity, interpretability, and predictive power. The GA optimization parameters were defined as follows: the number of generations per model size was set to 500, the population size (i.e., the number of models evolved in each generation) was set to 10, and a mutation rate of 20% was applied to promote descriptor diversity and avoid premature convergence. These settings were selected to ensure sufficient exploration of the descriptor space while maintaining computational efficiency and model stability.

### 3.3. 3D Field-Based QSAR Model

Prior to the development of the three-dimensional QSAR model, all molecular structures were subjected to geometry optimization using the MMFF94 force field as implemented in Marvin Sketch (Marvin 6.1.0, ChemAxon, Budapest, Hungary, 2013). Geometry optimization was performed to obtain energetically reasonable conformations suitable for subsequent field-based analysis, while avoiding overreliance on high-level quantum-mechanical calculations at this exploratory stage. For the construction of the 3D QSAR model, the same dataset partitioning strategy applied in the conformation-independent QSAR analysis was considered. Among the generated splits, the partition exhibiting the most stable and representative statistical performance in the conformation-independent modeling stage was selected as the basis for three-dimensional QSAR development. This choice was made to ensure internal consistency across modeling approaches while maintaining a clear separation between training and external test sets. Three-dimensional QSAR modeling was carried out using a field-based partial least squares (PLS) regression framework. The maximum number of PLS components was limited to six in order to prevent overparameterization and reduce the risk of overfitting. Steric and electrostatic field energy values were truncated at ±30.0 kcal·mol^−1^ to eliminate extreme grid-point contributions that could disproportionately influence model coefficients. A grid spacing of 1.0 Å was employed, with the grid extended by 3.0 Å beyond the spatial boundaries of the aligned training set molecules to ensure adequate sampling of the interaction fields. Grid variables exhibiting a standard deviation below 0.01 across the training set were excluded from the analysis, thereby reducing noise and improving model robustness. All three-dimensional field-based QSAR calculations were performed using Schrödinger Maestro (version 11.5.011), which provided an integrated environment for molecular alignment, field calculation, and PLS-based model construction. 

### 3.4. Validation of the Developed QSAR Models

A comprehensive validation strategy was applied to assess the quality, robustness, and predictive reliability of the developed conformation-independent and two-dimensional QSAR models. Multiple complementary statistical metrics were employed in order to evaluate both internal consistency and external predictive performance. These included the squared correlation coefficient (r^2^), root-mean-square error (RMSE), leave-one-out cross-validation coefficient (q^2^_LOO), leave-many-out cross-validation coefficient (q^2^_LMO), Fisher’s F-statistic, and the mean absolute error (MAE). In addition, y-scrambling tests were conducted to exclude chance correlations and confirm the statistical significance of the derived models [43,44,45,46]. To further strengthen model validation beyond conventional metrics, additional external validation criteria were applied. These included the R_m^2^ metrics and MAE-based validation parameters, the concordance correlation coefficient (CCC), and the index of ideality of correlation (IIC), all of which provide complementary insight into the balance between correlation strength, predictive accuracy, and systematic bias [47]. The combined use of these metrics ensured a rigorous and multifaceted evaluation of model performance, in line with current best practices in QSAR modeling. Definition of the applicability domain (AD) was considered an essential component of model validation, as reliable QSAR predictions can only be made for compounds that fall within the chemical space represented by the training data [48,49]. Accordingly, the AD was established prior to any predictive application of the developed QSAR models, ensuring that extrapolative predictions outside the model’s domain were avoided. In the present study, the applicability domain of the conformation-independent QSAR models was defined using the concept of “statistical defects” associated with molecular descriptors, denoted as d (A), which had already been employed during model development [33]. This approach quantifies deviations of descriptor values from the statistical characteristics of the training set and enables identification of compounds lying outside the reliable prediction space. All AD-related calculations were performed using the CORAL software package, in accordance with Equation (10) [50].

(10) $$d\left(A\right)=\frac{\left|P\left(A_{train}\right)-P\left(A_{test}\right)\right|}{N\left(A_{train}\right)-N\left(A_{trest}\right)}$$

In the equation above, P (A_train_) and P (A_calib_) define the probabilities of a conformation-independent attribute or descriptor (A) in the training and test sets, respectively, while N (A_train_) and N (A_calib_) define the frequency of a conformation-independent attribute or descriptor (A) appearing in the training set and the test set, respectively. The statistical SMILES defect (D) represents the sum of the defects, d (A) of all the attributes available in the SMILES notation molecules, and it is calculated according to Equation (11).

(11) $$D=defect\left(SMILES\right)=\sum_{k=1}^{NA}d\left(A\right)$$

A molecule is not classified in the defined AD, and is categorized as an outlier, provided its D > 2 × D_av_; where D_av_ is the average of the D calculated for the appropriate set (training set or test set) where the molecule is placed. 

For the GA-MLR QSAR models, a leverage-based approach was applied using the Williams plot (standardized residuals versus leverage values). The leverage (h_i_) for each compound was calculated from the hat matrix of the regression model. The warning leverage threshold (h*) was defined according to the commonly accepted criterion: h* = 3 (p + 1)/n; where p represents the number of model descriptors and n is the number of compounds in the training set. Compounds with leverage values exceeding h* were considered structurally influential, while those with standardized residuals outside ±3 standard deviations were identified as response outliers. Only compounds within both the leverage threshold and residual limits were considered reliably predicted by the model. The Williams plot analysis confirmed that the majority of compounds fell within the defined applicability domain, supporting the robustness and predictive reliability of the developed QSAR models.

### 3.5. Molecular Docking

Molecular docking studies were performed using the Molegro Virtual Docker (MVD 2013.6.0) software to provide a structure-based assessment of the binding potential of the studied compounds. Docking simulations were performed using the MolDock SE algorithm with default settings in MVD 6.0 (population size = 50, 10 runs per ligand, grid resolution = 0.30 Å, and 1500 iterations), followed by post-docking energy minimization through the Simplex evolution algorithm. The binding site was defined based on the co-crystallized ligand coordinates, and a grid with a resolution of 0.30 Å and a radius of 15 Å was used to encompass the active pocket. Ligands were geometry-optimized using the MMFF94 force field and docked using the guided differential evolution algorithm in MVD. For each ligand, 10 poses were generated, and the best-ranked conformations were selected based on MolDock Score and ReRank Score. Additional scoring functions, including hydrogen bonding energy, van der Waals interactions, steric clashes, and electrostatics, were analyzed for mechanistic interpretation. Docking calculations were performed using the same geometrically optimized ligand structures used to develop the three-dimensional QSAR model. Ligand flexibility was handled using the default MolDock conformational search protocol, which internally generates and optimizes multiple ligand conformations during docking. Docking studies were conducted for the selected template compound and the QSAR-guided designed molecules, with the aim of providing a qualitative, structure-based assessment of their binding behavior rather than a systematic evaluation of the entire QSAR dataset. The human serotonin transporter structure (Protein Data Bank identifier 6AWN) was selected as the molecular target for docking simulations due to its well-resolved ligand-binding site and relevance to SSRI binding. Although this structure corresponds to the S439T mutant bound to paroxetine, the mutation is located outside the primary binding pocket and does not directly affect the key residues involved in ligand recognition. Given the high structural similarity among available SERT crystal structures (including 6AWO, 6AWP, and 6AWQ), a single representative structure was employed for qualitative docking analysis. In the docking protocol, the receptor was treated as rigid, while ligands were allowed full conformational flexibility. This approach enables efficient sampling of ligand conformations within the binding site while maintaining a fixed protein geometry. Protocol validation was performed by redocking the co-crystallized ligand into the 6AWN binding site (cavity defined around the crystallographic ligand), yielding a heavy-atom RMSD of 1.72 Å. MVD evaluates ligand–receptor interactions by explicitly accounting for both hydrophobic and hydrophilic contributions. Hydrophobic interactions are primarily mediated by van der Waals and steric contacts, whereas hydrophilic interactions involve hydrogen bonding between ligand functional groups and amino acid residues within the active site. These interactions are quantitatively assessed using scoring functions that approximate relative binding energies [51]. In the present study, several scoring terms were calculated to characterize ligand–SERT interactions, including van der Waals (VdW), hydrogen bonding (Hbond), steric, and non-hydrogen bonding (NoHbond) contributions. In addition, composite scoring functions such as MolDock score, pose energy, and Rerank score were computed to provide an integrated evaluation of binding affinity and pose stability. These scoring metrics were used comparatively to assess the inhibitory potential of the studied ligands, rather than as absolute predictors of biological activity [34]. The overall docking protocol was validated in accordance with previously published methodologies to ensure reliability and reproducibility of the results [52,53]. For visualization and qualitative interpretation of ligand–receptor interactions, two-dimensional interaction diagrams were generated using Discovery Studio Client (version 20.1.0.19). These representations were employed to identify key amino acid residues involved in ligand binding and to support the interpretation of docking results in relation to QSAR-derived structure–activity trends.

### 3.6. InducedFit Docking and Binding Pose MetaDynamics (BPMD)

The induced-fit docking (IFD) protocol, developed by Schrödinger [24], was employed to model protein conformational changes associated with ligand binding. The IFD workflow, as described in previous studies [22,54], consists of an initial docking step in which each ligand is docked using a softened potential achieved by scaling van der Waals radii. This is followed by side-chain refinement of protein residues located within 5 Å of any ligand pose. The selected residues and the ligand are then subjected to energy minimization for each protein–ligand complex. At this stage, the resulting receptor conformations reflect structural adjustments induced by ligand binding.

In the final step, ligands are redocked into the induced-fit receptor structures using the Glide XP docking protocol (Glide, Schrödinger, LLC, 2024, New York, NY, USA). All IFD calculations were performed using the standard Schrödinger protocol with the OPLS4 force field.

Binding Pose Metadynamics (BPMD) is an automated enhanced-sampling protocol based on metadynamics designed to assess ligand binding stability. During BPMD simulations, ligands are biased to explore conformational space around their initial binding pose. For each system, ten independent metadynamics simulations of 10 ns each were performed. The collective variable (CV) was defined as the root-mean-square deviation (RMSD) of ligand heavy atoms relative to the starting pose. Prior to RMSD calculation, protein residues within 3 Å of the ligand were selected, and their Cα atoms were aligned to those of the first frame of the metadynamics trajectory. Following this alignment, the heavy-atom RMSD of the ligand was computed with respect to its initial conformation. The metadynamics bias applied is the same as that used in previous works [55,56,57]. The underlying principle of BPMD is that ligands not stably bound to the target binding site will exhibit larger RMSD values under the same biasing conditions compared to stably bound ligands. After completion of the simulations, ligand stability was evaluated using three metrics: PoseScore, PersistenceScore (PersScore), and CompositeScore (CompScore). PoseScore reflects the average RMSD of the ligand relative to its initial binding pose; a steep increase in this value indicates that the ligand is not confined to a well-defined energy minimum and may not have been accurately modeled. PersScore quantifies the persistence of hydrogen-bond interactions and is calculated over the final 2 ns of each simulation by assessing whether the same number of hydrogen bonds observed in the initial structure is maintained. The PersScore is averaged across the ten independent simulations and ranges from 0 to 1, where a value of 0 indicates either the absence of initial ligand–target interactions or complete loss of these interactions during the simulations, whereas a value of 1 indicates full retention of the interactions observed in the starting pose throughout the final 2 ns. The CompositeScore is defined as a linear combination of PoseScore and PersScore, with lower values corresponding to more stable ligand–protein complexes as presented in Equation (12).

CompScore = PoseScore − 5 × PersScore(12)

### 3.7. ADMET Prediction

In silico prediction of absorption, distribution, metabolism, excretion, and toxicity (ADMET) properties was performed using the DeepPK web platform (https://biosig.lab.uq.edu.au/deeppk/ (accessed on 22 February 2026)), a publicly available deep learning-based tool for pharmacokinetic and toxicity assessment. DeepPK employs neural network models trained on curated experimental datasets to predict a wide range of pharmacokinetic and safety-related endpoints relevant to drug discovery [58]. Canonical SMILES representations of all designed compounds were submitted to the DeepPK platform, and predicted ADMET parameters were retrieved without further manual intervention or model customization. The evaluated endpoints included parameters related to intestinal absorption, blood–brain barrier permeability, plasma protein binding, fraction unbound in human plasma, cytochrome P450 inhibition and substrate liability (CYP2D6 and CYP3A4), P-glycoprotein interaction, hERG channel blockade, mutagenicity, and drug-induced liver injury, as well as additional distribution- and clearance-related descriptors. The predicted ADMET data were used for comparative analysis to support compound prioritization and complement the QSAR and molecular docking analyses, rather than as definitive predictors of in vivo pharmacokinetic behavior. All ADMET results reported in this study represent in silico predictions and were interpreted within the known limitations of data-driven pharmacokinetic modeling.

## 4. Conclusions

The present study demonstrates the successful development and application of multiple complementary QSAR strategies for investigating and rationally optimizing serotonin transporter inhibitors. Robust and predictive quantitative structure–activity relationship models were established using conformation-independent Monte Carlo optimization, genetic algorithm-assisted multiple linear regression, and three-dimensional field-based analysis. The quality, stability, and predictive potential of all developed models were rigorously assessed using a comprehensive set of internal and external validation metrics, confirming their statistical robustness and well-defined applicability domains. The conformation-independent QSAR models, constructed from optimal descriptors derived from both SMILES notation and local molecular graph invariants, provided mechanistically interpretable insight into fragment-level contributions governing inhibitory activity. In parallel, GA–MLR modeling based on two-dimensional molecular descriptors yielded consistent structure–activity trends and converged on the same optimal data split as the Monte Carlo approach, reinforcing the reliability of the identified relationships across distinct modeling paradigms. Three-dimensional QSAR analysis further clarified the dominant interaction mechanisms underlying serotonin transporter inhibition. Field contribution analysis revealed that steric and hydrophobic interactions are the primary determinants of activity, while electrostatic and hydrogen-bond donor contributions play secondary roles. These findings provided a clear structural rationale for prioritizing shape complementarity and hydrophobic packing during ligand optimization. Fragment-level interpretation of SMILES-based descriptors enabled the identification of structural motifs exerting positive or negative effects on inhibitory potency. These insights were directly exploited in a computer-aided design strategy, leading to the generation of a focused series of novel candidate structures with systematically improved predicted pIC_50_ values relative to the parent scaffold. The observed structure–activity trends across the designed series were fully consistent with both the conformation-independent and three-dimensional QSAR results. Molecular docking studies and biased molecular dynamics simulations provided independent structure-based validation of the QSAR-guided design strategy. The docking and MD results showed qualitative agreement with predicted activities, particularly regarding steric and hydrophobic interactions, and supported the existence of multiple energetically favorable binding modes within the serotonin transporter cavity. This convergence of QSAR predictions and docking-derived interaction patterns strengthens confidence in the proposed computationally predicted inhibitory profiles of the designed compounds. Overall, the integrated computational framework presented in this study, combining interpretable QSAR modeling, three-dimensional field analysis, fragment-based molecular design, and docking and MD validation, provides a coherent and transferable strategy for the rational exploration of serotonin transporter inhibitors. The designed candidate structures represent computationally prioritized hypotheses for future synthesis and pharmacological evaluation; experimental validation of their predicted biological activity remains an essential and explicitly planned next step.

## Figures and Tables

**Table 1:** The statistical quality of the QSAR models developed with the Monte Carlo optimization method for serotonin transport inhibition.

	Run	Training Set							Test Set						
		r^2^	CCC	IIC	q^2^	s	MAE	F	r^2^	CCC	IIC	q^2^	s	MAE	F
**Split 1**	1	0.9351	0.9665	0.9161	0.9283	0.142	0.099	504	0.8898	0.7481	0.9433	0.8516	0.511	0.408	89
	2	0.9542	0.9766	0.9255	0.9497	0.120	0.095	729	0.8837	0.7797	0.9401	0.8495	0.492	0.387	84
	3	0.9660	0.9827	0.9311	0.9621	0.103	0.077	995	0.8661	0.8769	0.9306	0.7574	0.390	0.322	71
	Av	0.9518	0.9753	0.9242	0.9467	0.122	0.090	743	0.8799	0.8016	0.9380	0.8195	0.464	0.372	81
**Split 2**	1	0.8882	0.9408	0.8011	0.8773	0.223	0.169	278	0.9076	0.9058	0.9520	0.8844	0.295	0.244	108
	2	0.8742	0.9329	0.8858	0.8627	0.237	0.176	243	0.9085	0.9255	0.9531	0.8835	0.265	0.221	109
	3	0.8963	0.9453	0.8047	0.8833	0.215	0.174	302	0.8816	0.9099	0.9386	0.8497	0.291	0.241	82
	Av	0.8862	0.9397	0.8305	0.8744	0.225	0.173	274	0.8992	0.9137	0.9479	0.8725	0.284	0.235	100
**Split 3**	1	0.8365	0.9109	0.7403	0.8218	0.307	0.242	184	0.8734	0.9197	0.9345	0.8277	0.133	0.109	69
	2	0.7674	0.8684	0.7092	0.7396	0.366	0.282	119	0.9034	0.9290	0.9503	0.8453	0.124	0.092	93
	3	0.8574	0.9232	0.9259	0.8406	0.286	0.233	216	0.8738	0.9321	0.9340	0.8334	0.129	0.105	69
	Av	0.8204	0.9008	0.7918	0.8007	0.320	0.252	173	0.8835	0.9269	0.9396	0.8355	0.129	0.102	77

r^2^—Correlation coefficient; CCC—Concordance correlation coefficient; IIC—Index of ideality of correlation; q^2^—Cross-validated correlation coefficient; s—Standard error of estimation; MAE—Mean absolute error; F—Fischer ratio; Av—Average value for statistical parameters obtained from three independent Monte Carlo optimization runs.

**Table 2:** The statistical quality of the QSAR models developed with the GA-MLR method for serotonin transporter inhibition.

Fitting Criteria										
S	R^2^	R^2^_adj_	R^2^−R^2^_adj_	LOF	RMSE_tr_	MAE_tr_	RSS_tr_	CCC_tr_	s	F
**1**	0.8464	0.8216	0.0248	0.0972	0.2275	0.1799	1.9144	0.9168	0.2485	34
**2**	0.8804	0.8611	0.0193	0.0973	0.2277	0.1766	1.9175	0.9364	0.2487	46
**3**	0.8719	0.8519	0.0200	0.1280	0.2637	0.2098	2.6419	0.9316	0.2873	44
**Internal Validation Criteria**							**External Validation Criteria**			
**S**	**Q^2^_loo_**	**R^2^-Q^2^_loo_**	**RMSE_cv_**	**MAE_cv_**	**PRESS_cv_**	**CCC_cv_**	**RMSE_ext_**	**MAE_ext_**	**PRESS_ext_**	
**1**	0.7781	0.0683	0.2734	0.2179	2.7653	0.8780	0.8720	0.6339	9.8857	
**2**	0.8511	0.0293	0.2540	0.2057	2.3868	0.9212	0.4369	0.3939	2.4813	
**3**	0.8266	0.0453	0.3068	0.2470	3.5762	0.9072	0.5697	0.4503	3.8946	
**Predictions by LOO**										
**S**	**R′^2^**	**R′^2^_o_**	**k′**	**C_los_′**	**r′^2^_m_**	**R^2^**	**R^2^_o_**	**k**	**C_los_**	**r^2^_m_**
**1**	0.7789	0.7287	1.0002	0.0645	0.6044	0.7789	0.7785	0.9986	0.0005	0.7629
**2**	0.8516	0.8328	0.9999	0.0221	0.7347	0.8516	0.8512	0.9990	0.0005	0.8347
**3**	0.8274	0.8002	1.0002	0.0328	0.6910	0.8274	0.8270	0.9982	0.0005	0.8105

S—Molecular database split; R^2^—The Coefficient of Determination; R^2^_adj_—adjusted R^2^; LOF—Lack-of-fit; LOO—Leave One Out; RMSE—Root Mean Square Error; RMSE_tr_—for training set, RMSE_cv_—for internal validation; RMSE_ext_—for external validation; MAE—Mean Absolute Error; MAE_tr_—for training set, MAE_cv_—for internal validation; RSS—Residual Sum of Squares; CCC—The Concordance Correlation Coefficient; CCC_tr_—for training set, CCC_cv_—for internal validation; s—Standard Deviation; PRESS—The Predicted Residual Error Sum of Squares; PRESS_cv_—for internal validation; PRESS_ext_—for external validation; r^2^_m_ [30,31,32]; In predictions by LOO “**′**“ indicates Exp (x) vs. Pred (y) while without indicates Pred (x) vs. Exp (y).

**Table 3:** An example of the DCW (1, 10) calculation.

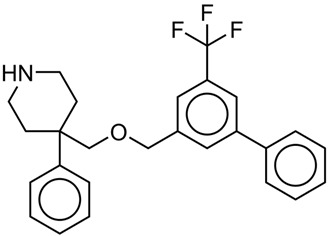				SMILES notation: Cn1nnc(n1)c1cc(COCC2(CCNCC2)c2ccccc2)cc(c1)C(F)(F)F DCW = 111.17841 Prediction for EndPoint = 8.0321			
SA (CW)	CW	SA (CW)	CW	SA (CW)	CW	SA (CW)	CW
C...........	0.3447	1...........	−0.8026	c...c.......	0.4688	C...C...(...	−0.8527
n...........	0.0186	(...........	0.269	c...c.......	0.4688	N...C...C...	2.1912
1...........	−0.8026	C...........	0.3447	c...c.......	0.4688	C...N...C...	2.1254
n...........	0.0186	(...........	0.269	c...c.......	0.4688	N...C...C...	2.1912
n...........	0.0186	F...........	0.1799	c...2.......	2.4377	C...C...2...	2.2071
c...........	−0.4471	(...........	0.269	2...(.......	−0.1962	C...2...(...	0.4969
(...........	0.269	(...........	0.269	c...(.......	0.3964	c...(...2...	−0.124
n...........	0.0186	F...........	0.1799	c...c.......	0.4688	2...c...(...	−0.6234
1...........	−0.8026	(...........	0.269	c...(.......	0.3964	c...2...c...	−0.8324
(...........	0.269	F...........	0.1799	c...(.......	0.3964	c...c...2...	−0.6698
c...........	−0.4471	n...C.......	0.2397	c...1.......	0.1739	c...c...c...	−0.8924
1...........	−0.8026	n...1.......	0.4141	1...(.......	0.1163	c...c...c...	−0.8924
c...........	−0.4471	n...1.......	0.4141	C...(.......	−0.7408	c...c...c...	−0.8924
c...........	−0.4471	n...n.......	−0.7738	C...(.......	−0.7408	c...c...2...	−0.6698
(...........	0.269	n...c.......	0.0923	F...(.......	−0.8252	c...2...(...	−2.0438
C...........	0.3447	c...(.......	0.3964	F...(.......	−0.8252	c...(...2...	−0.124
O...........	0.6288	n...(.......	2.0629	(...(.......	−1.4187	c...c...(...	0.9158
C...........	0.3447	n...1.......	0.4141	F...(.......	−0.8252	c...c...(...	0.9158
C...........	0.3447	1...(.......	0.1163	F...(.......	−0.8252	c...(...c...	−0.856
2...........	2.3436	c...(.......	0.3964	F...(.......	−0.8252	1...c...(...	0.1144
(...........	0.269	c...1.......	0.1739	C...n...1...	−0.9283	c...1...(...	1.4353
C...........	0.3447	c...1.......	0.1739	n...1...n...	0.2246	C...(...1...	1.0971
C...........	0.3447	c...c.......	0.4688	n...n...1...	0.1801	(...C...(...	0.1113
N...........	0.295	c...(.......	0.3964	n...n...c...	−1.0518	F...(...C...	2.1949
C...........	0.3447	C...(.......	−0.7408	n...c...(...	0.2283	(...F...(...	0.1479
C...........	0.3447	O...C.......	−0.7979	n...(...c...	0.5503	F...(...(...	0.1855
2...........	2.3436	O...C.......	−0.7979	1...n...(...	2.8307	F...(...(...	0.1855
(...........	0.269	C...C.......	0.1772	n...1...(...	3.551	(...F...(...	0.1479
c...........	−0.4471	C...2.......	0.1987	c...(...1...	0.2971	F...(...F...	0.4795
2...........	2.3436	2...(.......	−0.1962	1...c...(...	0.1144	Cmax.2......	2.1757
c...........	−0.4471	C...(.......	−0.7408	c...1...c...	0.0403	Nmax.1......	0.3841
c...........	−0.4471	C...C.......	0.1772	c...c...1...	2.3309	Omax.1......	0.5766
c...........	−0.4471	N...C.......	1.0573	c...c...(...	0.9158	Smax.0......	0.8094
c...........	−0.4471	N...C.......	1.0573	c...(...C...	0.426	NOSP11000000	5.7413
2...........	2.3436	C...C.......	0.1772	O...C...(...	−0.2246	HALO10000000	−0.6663
(...........	0.269	C...2.......	0.1987	C...O...C...	0.1366	BOND00000000	0.5132
c...........	−0.4471	2...(.......	−0.1962	O...C...C...	1.2334	++++F---N===	−0.2205
c...........	−0.4471	c...(.......	0.3964	C...C...2...	2.2071	++++F---O===	−0.9908
(...........	0.269	c...2.......	2.4377	C...2...(...	0.4969	++++N---O===	6.1527
c...........	−0.4471	c...2.......	2.4377	C...(...2...	−1.0604	$00011001000	−0.6679

**Table 4:** A list of all the designed molecules with their SMILES notation and calculated activities.

Molecule	SMILES Notation	pIC_50_ (Calc.)
**A**	FC(c1cc(COCC2(CCNCC2)c2ccccc2)cc(c1)c1ccccc1)(F)F	7.8952
**A1**	Fc1ccccc1c1cc(COCC2(CCNCC2)c2ccccc2)cc(c1)C(F)(F)F	8.0215
**A2**	Fc1cccc(c1)c1cc(COCC2(CCNCC2)c2ccccc2)cc(c1)C(F)(F)F	7.9310
**A3**	Fc1ccc(cc1)c1cc(COCC2(CCNCC2)c2ccccc2)cc(c1)C(F)(F)F	8.1855
**A4**	CC(c1ccccc1c1cc(COCC2(CCNCC2)c2ccccc2)cc(c1)C(F)(F)F)C	8.0070
**A5**	CC(C)c1cccc(c1)c1cc(COCC2(CCNCC2)c2ccccc2)cc(c1)C(F)(F)F	7.6785
**A6**	CC(c1ccc(cc1)c1cc(COCC2(CCNCC2)c2ccccc2)cc(c1)C(F)(F)F)C	8.0295
**A7**	N#Cc1ccc(cc1)c1cc(COCC2(CCNCC2)c2ccccc2)cc(c1)C(F)(F)F	8.1943
**A8**	CN(c1ccc(cc1)c1cc(COCC2(CCNCC2)c2ccccc2)cc(c1)C(F)(F)F)C	8.4814

**Table 5:** Score values (kcal/mol) for all the computer-aided designed compounds.

Molecule	HBond	NoHBond90	Steric	VdW	Energy	MolDock	Rerank	pIC_50_ (Calc.)
**A**	0	0	−175.949	−42.2977	−153.22	−149.929	−98.698	7.8952
**A1**	0	−0.69399	−181.955	−22.7734	−157.652	−154.178	−117.876	8.0215
**A2**	−2.40454	−2.40454	−181.522	20.5607	−159.86	−152.977	−95.0434	7.931
**A3**	0	−2.5	−203.228	−43.7415	−177.416	−174.068	−139.04	8.1855
**A4**	−2.19821	−2.5	−182.575	−41.1272	−173.611	−164.111	−121.914	8.007
**A5**	−1.09053	−6.1829	−181.313	25.7723	−158.802	−156.679	−89.5642	7.6785
**A6**	0	0	−189.35	−50.3446	−171.667	−169.208	−138.993	8.0295
**A7**	0	−0.20938	−182.255	−48.7535	−163.942	−161.211	−132.397	8.1943
**A8**	−0.37688	−4.80842	−194.602	28.4043	−176.14	−172.675	−125.456	8.4814

**Table 6:** CompScore for copounds **A**–**A8**.

Ligand	CompScore
**A**	1.521
**A1**	0.010
**A2**	0.805
**A3**	1.033
**A4**	−2.649
**A5**	1.070
**A6**	0.479
**A7**	−0.930
**A8**	0.730

## Data Availability

The original contributions presented in this study are included in the article/Supplementary Material. Further inquiries can be directed to the corresponding author.

## Supplementary Materials

The following supporting information can be downloaded at https://www.mdpi.com/article/10.3390/ph19030444/s1. Table S1: The SMILES notation of the studied molecules, calculated values for the DCW, experimental data (Ac)—expr, the values of Ac calculated; Table S2: Y-randomization of the best QSAR model (best optimization run) for three independent splits; Table S3: The list of SAks together with their correlation weights for the three runs of the Monte Carlo optimization; Table S4: in silico ADMET profile of the designed serotonin transporter inhibitors; Figure S1: **Above left**) Graphical representation of developed QSAR model with GA-MLR method for split 1; **Above right**) Difference between experimental and calculated pIC50 values; **Below)** Graphical representation of applicability domain established for split 1; Figure S2: **Above left**) Graphical representation of developed QSAR model with GA-MLR method for split 2; **Above right**) Difference between experimental and calculated pIC_50_ values; **Below)** Graphical representation of applicability domain established for split 2; Figure S3: **Above left**) Graphical representation of developed QSAR model with GA-MLR method for split 3; **Above right**) Difference between experimental and calculated pIC50 values; **Below)** Graphical representation of applicability domain established for split 3; Figure S4: Correlation analysis between docking scores and calculated pIC50 values for compounds **A**–**A8**; Figure S5: Two-dimensional representation of the interaction between molecule **A** and amino acids inside serotonin transporter binding pocket; Figure S6: Two-dimensional representation of the interaction between molecule **A1** and amino acids inside serotonin transporter binding pocket; Figure S7: Two-dimensional representation of the interaction between molecule **A2** and amino acids inside serotonin transporter binding pocket; Figure S8: Two-dimensional representation of the interaction between molecule **A3** and amino acids inside serotonin transporter binding pocket; Figure S9: Two-dimensional representation of the interaction between molecule **A4** and amino acids inside serotonin transporter binding pocket; Figure S10. Two-dimensional representation of the interaction between molecule **A5** and amino acids inside serotonin transporter binding pocket; Figure S11: Two-dimensional representation of the interaction between molecule **A6** and amino acids inside serotonin transporter binding pocket; Figure S12: Two-dimensional representation of the interaction between molecule **A7** and amino acids inside serotonin transporter binding pocket; Figure S13: Two-dimensional representation of the interaction between molecule **A8** and amino acids inside serotonin transporter binding pocket. Figure S14. Structural diversity analysis of the QSAR dataset (n = 50 compounds). (**A**) Pairwise Tanimoto similarity matrix computed from SMILES-based structural fingerprints for all 50 compounds (1225 unique pairs). Color scale ranges from blue (low similarity) to red (high similarity). (**B**) Frequency distribution of pairwise Tanimoto similarity values. Mean similarity = 0.700 (SD = 0.158); 41.8% of compound pairs exhibit Tanimoto similarity below 0.70, indicating meaningful structural variation within the dataset. (**C**) Composition of the dataset by substituent class, illustrating the systematic exploration of six distinct structural subgroups. (**D**) Cumulative distribution of pairwise similarities; 11.8% of pairs fall below Tanimoto = 0.50, confirming the presence of structurally distinct compound clusters, particularly among heterocyclic derivatives.
