# Supplementary material for: QSAR-Guided Design of Serotonin Transporter Inhibitors Supported by Molecular Docking and Biased Molecular Dynamics
Source: Pharmaceuticals (Basel). 2026 Mar 10;19(3):444. doi: 10.3390/ph19030444 (PMC13029183; doi:10.3390/ph19030444)
Supplement: Supplementary file 1 [file pharmaceuticals-19-00444-s001.zip › Supplementary material R2.pdf]

Table S1. The SMILES notation of the studied molecules, calculated values for the DCW, experimental data (Ac) – expr, the values of Ac calculated with the application of CORAL software – calc, the difference between expr and calc – diff for the built QSPR model

			Split 1				Split 2				Split 3			
	SMILES notation	Expr.	DCW	Calc.	Diff	Set	DCW	Calc.	Diff	Set	DCW	Calc.	Diff	Set
1	COc1ccc(cc1COCC1(CCNCC1)c1ccccc1)n1nnnc1C	7.432	214.7311	7.4187	0.0133	Tr	93.80202	7.3604	0.0716	Tr	199.1564	7.5399	-0.1079	Tr
2	Cc1nnnn1c1cc(COCC2(CCNCC2)c2ccccc2)cc(c1)C(F)(F)F	7.155	214.7677	7.4197	-0.2647	Ts	90.94673	7.25	-0.095	Tr	186.725	7.097	0.058	Tr
3	FC(c1cc(COCC2(CCNCC2)c2ccccc2)cc(c1)n1nnnc1C(F)(F)F)(F)F	7.244	206.6637	7.2106	0.0334	Tr	95.85697	7.4398	-0.1958	Ts	192.7047	7.31	-0.066	Tr
4	FC(c1cc(COCC2(CCNCC2)c2ccccc2)cc(c1)C1NNNN1)(F)F	8.066	240.128	8.0738	-0.0078	Tr	112.0027	8.0639	0.0021	Tr	209.2339	7.899	0.167	Ts
5	Cn1nnnc1c1cc(COCC2(CCNCC2)c2ccccc2)cc(c1)C(F)(F)F	7.301	209.3935	7.281	0.02	Tr	96.57472	7.4676	-0.1666	Tr	191.6637	7.2729	0.0281	Ts
6	Cn1nnc(n1)c1cc(COCC2(CCNCC2)c2ccccc2)cc(c1)C(F)(F)F	8.018	236.7095	7.9856	0.0324	Tr	111.1784	8.0321	-0.0141	Tr	209.2022	7.8979	0.1201	Tr
7	FC(c1cc(COCC2(CCNCC2)c2ccccc2)cc(c1)c1ccccc1)(F)F	8.432	237.5313	8.0068	0.4252	Tr	107.6381	7.8952	0.5368	Tr	206.9497	7.8177	0.6143	Tr
8	FC(c1cc(COCC2(CCNCC2)c2ccccc2)cc(c1)c1ccncc1)(F)F	7.796	237.1874	7.998	-0.202	Tr	117.6827	8.2835	-0.4875	Tr	219.9734	8.2817	-0.4857	Tr
9	FC(c1cc(COCC2(CCNCC2)c2ccccc2)cc(c1)c1ccccn1)(F)F	8.131	234.5771	7.9306	0.2004	Ts	110.6729	8.0125	0.1185	Tr	213.5489	8.0528	0.0782	Ts
10	FC(c1cc(COCC2(CCNCC2)c2ccccc2)cc(c1)c1ccco1)(F)F	7.745	231.1048	7.8411	-0.0961	Tr	106.2998	7.8435	-0.0985	Tr	208.0585	7.8572	-0.1122	Tr
11	FC(c1cc(COCC2(CCNCC2)c2ccccc2)cc(c1)c1nccs1)(F)F	8.387	251.1674	8.3586	0.0284	Tr	120.7323	8.4014	-0.0144	Tr	227.4527	8.5483	-0.1613	Tr
12	FC(c1cc(COCC2(CCNCC2)c2ccccc2)cc(c1)c1ccc2c(c1)cccc2)(F)F	6.959	224.5306	7.6715	-0.7125	Ts	87.18382	7.1045	-0.1455	Tr	187.9395	7.1402	-0.1812	Tr
13	FC(c1cc(COCC2(CCNCC2)c2ccccc2)cc(c1)c1cccc2c1cccc2)(F)F	6.921	210.8469	7.3185	-0.3975	Ts	84.56284	7.0032	-0.0822	Tr	186.8234	7.1005	-0.1795	Tr
14	Clc1ccccc1c1cc(COCC2(CCNCC2)c2ccccc2)cc(c1)C(F)(F)F	8.194	250.1999	8.3336	-0.1396	Tr	121.8313	8.4439	-0.2499	Tr	217.5093	8.1939	0.0001	Ts
15	Fc1ccccc1c1cc(COCC2(CCNCC2)c2ccccc2)cc(c1)C(F)(F)F	8.119	243.2287	8.1538	-0.0348	Tr	110.9058	8.0215	0.0975	Ts	214.1857	8.0755	0.0435	Ts
16	Cc1ccccc1c1cc(COCC2(CCNCC2)c2ccccc2)cc(c1)C(F)(F)F	7.886	235.7518	7.9609	-0.0749	Tr	107.4087	7.8863	-0.0003	Ts	209.1599	7.8964	-0.0104	Ts
17	O=N(=O)c1ccccc1c1cc(COCC2(CCNCC2)c2ccccc2)cc(c1)C(F)(F)F	8.456	254.9846	8.457	-0.001	Tr	120.6868	8.3996	0.0564	Tr	235.1894	8.824	-0.368	Tr
18	OCc1ccccc1c1cc(COCC2(CCNCC2)c2ccccc2)cc(c1)C(F)(F)F	8.377	230.0913	7.8149	0.5621	Ts	117.5378	8.2779	0.0991	Tr	216.4525	8.1563	0.2207	Ts
19	Cc1cccc(c1)c1cc(COCC2(CCNCC2)c2ccccc2)cc(c1)C(F)(F)F	7.854	226.8457	7.7312	0.1228	Tr	111.0961	8.0289	-0.1749	Tr	204.6167	7.7345	0.1195	Tr
20	N#Cc1cccc(c1)c1cc(COCC2(CCNCC2)c2ccccc2)cc(c1)C(F)(F)F	8	254.2293	8.4375	-0.4375	Tr	115.6747	8.2059	-0.2059	Ts	211.6765	7.9861	0.0139	Ts
21	Fc1cccc(c1)c1cc(COCC2(CCNCC2)c2ccccc2)cc(c1)C(F)(F)F	8.201	242.1598	8.1262	0.0748	Tr	108.5649	7.931	0.27	Ts	204.6285	7.7349	0.4661	Tr
22	Nc1cccc(c1)c1cc(COCC2(CCNCC2)c2ccccc2)cc(c1)C(F)(F)F	7.523	225.7248	7.7023	-0.1793	Ts	109.39	7.9629	-0.4399	Tr	201.1052	7.6094	-0.0864	Ts
23	O=N(=O)c1cccc(c1)c1cc(COCC2(CCNCC2)c2ccccc2)cc(c1)C(F)(F)F	8.377	249.0817	8.3048	0.0722	Tr	110.8191	8.0182	0.3588	Tr	231.2033	8.6819	-0.3049	Tr
24	Oc1cccc(c1)c1cc(COCC2(CCNCC2)c2ccccc2)cc(c1)C(F)(F)F	7.569	217.1179	7.4803	0.0887	Tr	108.8106	7.9405	-0.3715	Ts	200.444	7.5858	-0.0168	Tr
25	FC(c1cc(COCC2(CCNCC2)c2ccccc2)cc(c1)c1ccc2c(c1)OCO2)(F)F	7.347	206.0885	7.1958	0.1512	Tr	93.47683	7.3478	-0.0008	Ts	190.588	7.2346	0.1124	Tr
26	FC(c1cc(COCC2(CCNCC2)c2ccccc2)cc(c1)c1ccc(cc1)C(F)(F)F)(F)F	7.444	216.5161	7.4648	-0.0208	Tr	93.84774	7.3621	0.0819	Tr	197.7788	7.4909	-0.0469	Tr
27	Clc1ccc(cc1)c1cc(COCC2(CCNCC2)c2ccccc2)cc(c1)C(F)(F)F	8.745	266.7369	8.7602	-0.0152	Tr	121.1955	8.4193	0.3257	Ts	221.7439	8.3448	0.4002	Tr
28	COC(=O)c1ccc(cc1)c1cc(COCC2(CCNCC2)c2ccccc2)cc(c1)C(F)(F)F	7.602	225.472	7.6958	-0.0938	Tr	108.8718	7.9429	-0.3409	Ts	214.2831	8.079	-0.477	Tr
29	Fc1ccc(cc1)c1cc(COCC2(CCNCC2)c2ccccc2)cc(c1)C(F)(F)F	8.824	261.0998	8.6148	0.2092	Tr	115.1473	8.1855	0.6385	Tr	211.9844	7.9971	0.8269	Tr
30	Cc1ccc(cc1)c1cc(COCC2(CCNCC2)c2ccccc2)cc(c1)C(F)(F)F	8.194	238.8313	8.0404	0.1536	Ts	113.5305	8.123	0.071	Tr	212.3056	8.0085	0.1855	Ts

31	CN(c1ccc(cc1)c1cc(COCC2(CCNCC2)c2ccccc2)cc(c1)C(F)(F)F)C	8.456	234.3139	7.9238	0.5322	Ts	122.8014	8.4814	-0.0254	Tr	222.777	8.3816	0.0744	Tr
32	O=N(=O)c1ccc(cc1)c1cc(COCC2(CCNCC2)c2ccccc2)cc(c1)C(F)(F)F	8.137	244.891	8.1967	-0.0597	Tr	118.943	8.3322	-0.1952	Tr	232.3197	8.7217	-0.5847	Tr
33	CCOc1ccc(cc1)c1cc(COCC2(CCNCC2)c2ccccc2)cc(c1)C(F)(F)F	7.854	231.6127	7.8542	-0.0002	Ts	104.2915	7.7658	0.0882	Tr	207.6519	7.8427	0.0113	Tr
34	Oc1ccc(cc1)c1cc(COCC2(CCNCC2)c2ccccc2)cc(c1)C(F)(F)F	7.569	225.0981	7.6861	-0.1171	Ts	108.3618	7.9232	-0.3542	Ts	203.0068	7.6771	-0.1081	Tr
35	COc1ccc(cc1)c1cc(COCC2(CCNCC2)c2ccccc2)cc(c1)C(F)(F)F	7.824	240.2804	8.0777	-0.2537	Tr	104.8843	7.7888	0.0352	Tr	206.8806	7.8152	0.0088	Tr
36	N#Cc1ccc(cc1)c1cc(COCC2(CCNCC2)c2ccccc2)cc(c1)C(F)(F)F	8.398	247.1699	8.2555	0.1425	Tr	115.3751	8.1943	0.2037	Tr	208.164	7.8609	0.5371	Tr
37	N#Cc1ccc(cc1F)c1cc(COCC2(CCNCC2)c2ccccc2)cc(c1)C(F)(F)F	8.051	239.9457	8.0691	-0.0181	Tr	113.6657	8.1282	-0.0772	Tr	227.506	8.5502	-0.4992	Tr
38	N#Cc1c(F)cc(cc1F)c1cc(COCC2(CCNCC2)c2ccccc2)cc(c1)C(F)(F)F	8.155	244.4208	8.1845	-0.0295	Tr	122.998	8.489	-0.334	Tr	218.7776	8.2391	-0.0841	Ts
39	N#Cc1cc(F)c(cc1F)c1cc(COCC2(CCNCC2)c2ccccc2)cc(c1)C(F)(F)F	8.432	250.8923	8.3515	0.0805	Tr	116.5527	8.2398	0.1922	Ts	214.1365	8.0737	0.3583	Tr
40	N#Cc1c(F)c(F)c(c(c1F)F)c1cc(COCC2(CCNCC2)c2ccccc2)cc(c1)C(F)(F)F	7.328	209.207	7.2762	0.0518	Tr	90.63488	7.2379	0.0901	Tr	198.4698	7.5155	-0.1875	Ts
41	N#Cc1ccc(cc1Cl)c1cc(COCC2(CCNCC2)c2ccccc2)cc(c1)C(F)(F)F	8.509	255.3329	8.466	0.043	Tr	130.8664	8.7931	-0.2841	Tr	231.222	8.6826	-0.1736	Tr
42	N#Cc1ccc(cc1C)c1cc(COCC2(CCNCC2)c2ccccc2)cc(c1)C(F)(F)F	8.569	236.3662	7.9768	0.5922	Ts	120.0562	8.3752	0.1938	Tr	221.8376	8.3482	0.2208	Tr
43	N#Cc1ccc(nc1)c1cc(COCC2(CCNCC2)c2ccccc2)cc(c1)C(F)(F)F	8.398	254.7264	8.4504	-0.0524	Tr	121.4547	8.4293	-0.0313	Tr	219.5604	8.267	0.131	Tr
44	N#Cc1ccc(cn1)c1cc(COCC2(CCNCC2)c2ccccc2)cc(c1)C(F)(F)F	8	241.6969	8.1143	-0.1143	Tr	114.6471	8.1662	-0.1662	Ts	216.3877	8.154	-0.154	Tr
45	N#Cc1ccc(cc1)c1cc(COCC2(CCNCC2)c2ccccc2)cc(c1)C(F)(F)F	8.398	247.1699	8.2555	0.1425	Tr	115.3751	8.1943	0.2037	Tr	208.164	7.8609	0.5371	Tr
46	N#Cc1ccc(cc1)c1cc(COCC2(CCN(CC2)C)c2ccccc2)cc(c1)C(F)(F)F	8.022	234.2329	7.9218	0.1002	Tr	102.212	7.6855	0.3365	Tr	191.0926	7.2526	0.7694	Tr
47	CCN1CCC(CC1)(COCc1cc(cc(c1)C(F)(F)F)c1ccc(cc1)C#N)c1ccccc1	6.377	179.8991	6.5202	-0.1432	Tr	73.22278	6.5649	-0.1879	Tr	171.1304	6.5413	-0.1643	Tr
48	N#Cc1ccc(cc1)c1cc(COCC2(CCN(CC2)C(C)C)c2ccccc2)cc(c1)C(F)(F)F	6.347	174.6516	6.3849	-0.0379	Tr	71.33949	6.4921	-0.1451	Tr	181.2114	6.9005	-0.5535	Tr
49	N#Cc1ccc(cc1)c1cc(COCC2(CCN(CC2)C2CC2)c2ccccc2)cc(c1)C(F)(F)F	5.678	185.583	6.6669	-0.9889	Ts	59.49567	6.0343	-0.3563	Ts	161.8301	6.2098	-0.5318	Tr
50	CCCCN1CCC(CC1)(COCc1cc(cc(c1)C(F)(F)F)c1ccc(cc1)C#N)c1ccccc1	6.097	186.933	6.7017	-0.6047	Ts	59.49556	6.0342	0.0628	Tr	161.17	6.1863	-0.0893	Tr

Table S2. Y-randomization of the best QSAR model (best optimization run) for three independent splits

	Split 1		Split 2		Split 3	
Run	Training	Test	Training	Test	Training	Test
0	0.9351	0.8898	0.8742	0.9085	0.7674	0.9034
1	0.013	0.0053	0.0589	0.0088	0.0331	0.0819
2	0.0004	0.1177	0.0194	0.4316	0.0249	0.3628
3	0.0374	0.0157	0.0061	0.0837	0.0001	0.1988
4	0.0262	0.0292	0.0017	0.0002	0.0151	0.1632
5	0.0253	0.1709	0.0212	0	0.1138	0.1601
6	0.0744	0.0109	0.0103	0.014	0.0023	0.0999
7	0.0155	0.2178	0.0001	0.1951	0.004	0.1656
8	0.002	0.1523	0.0018	0.0004	0.0108	0.5553
9	0.0196	0.0727	0.0554	0.1437	0	0.4705
10	0.0129	0.1912	0.0709	0.0213	0.015	0.0219
R_r^2	0.0227	0.0984	0.0246	0.0899	0.0219	0.228
${}^{C}R_p^2$	0.9237	0.8392	0.8618	0.8624	0.7564	0.7811
${}^{C}R_p^2 = R \times (R^2-R_r^2)^{1/2}$ should be > 0.5						

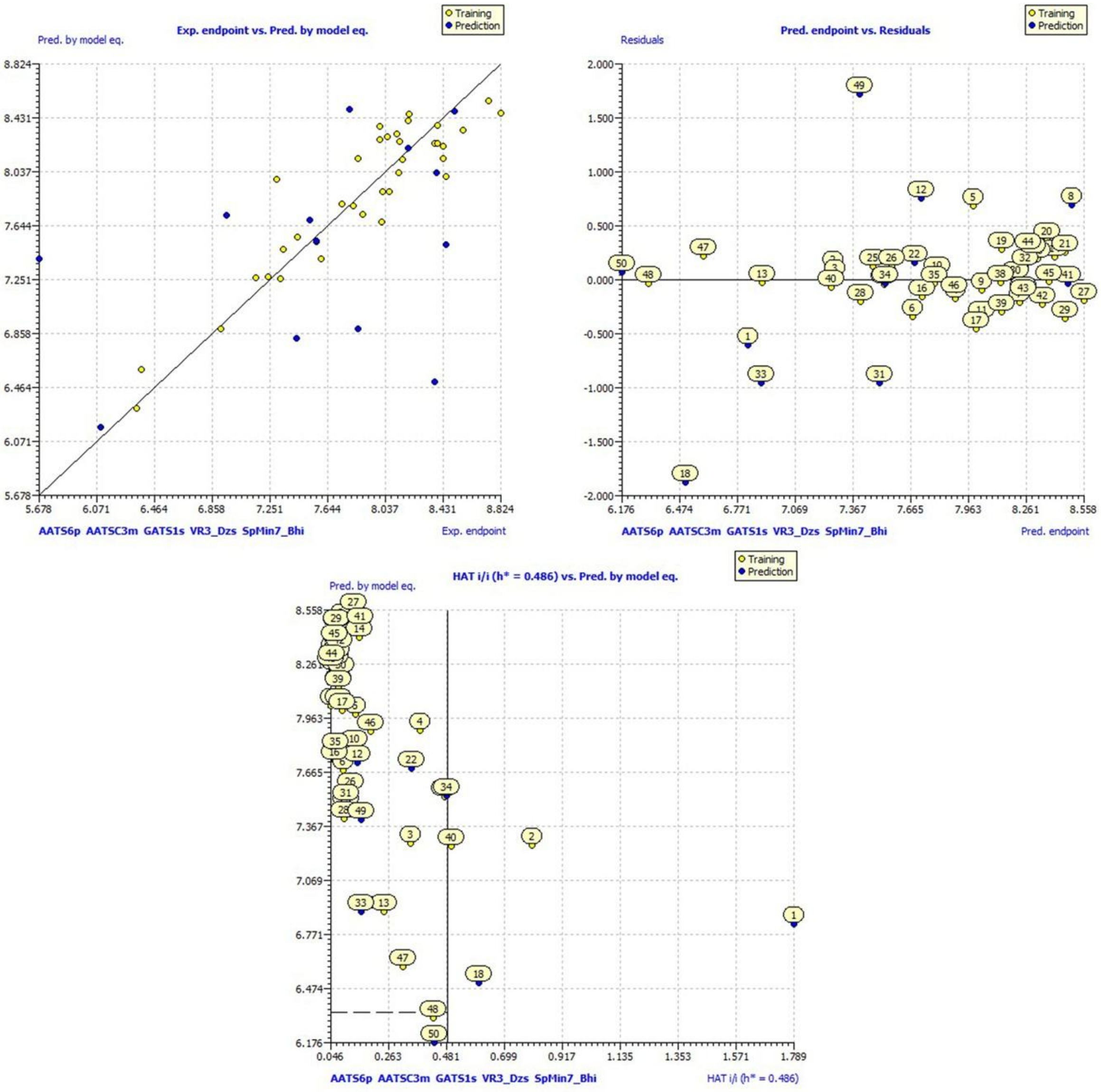

Figure S1. Above left) Graphical representation of developed QSAR model with GA-MLR method for split 1; Above right) Difference between experimental and calculated pIC50 values; Bellow) Graphical representation of applicability domain established for split 1.

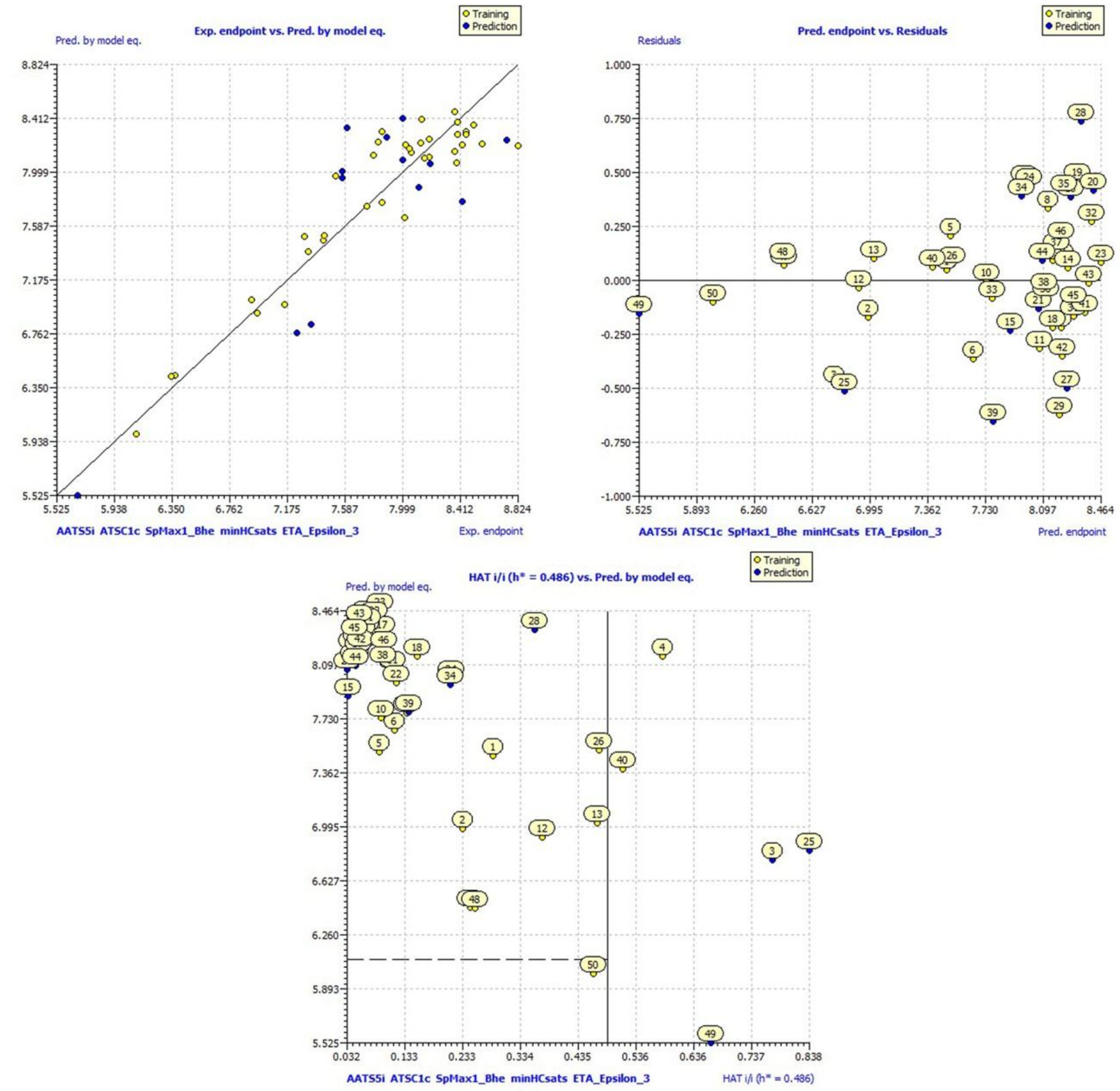

Figure S2. Above left) Graphical representation of developed QSAR model with GA-MLR method for split 2; Above right) Difference between experimental and calculated pIC50 values; Bellow) Graphical representation of applicability domain established for split 2.

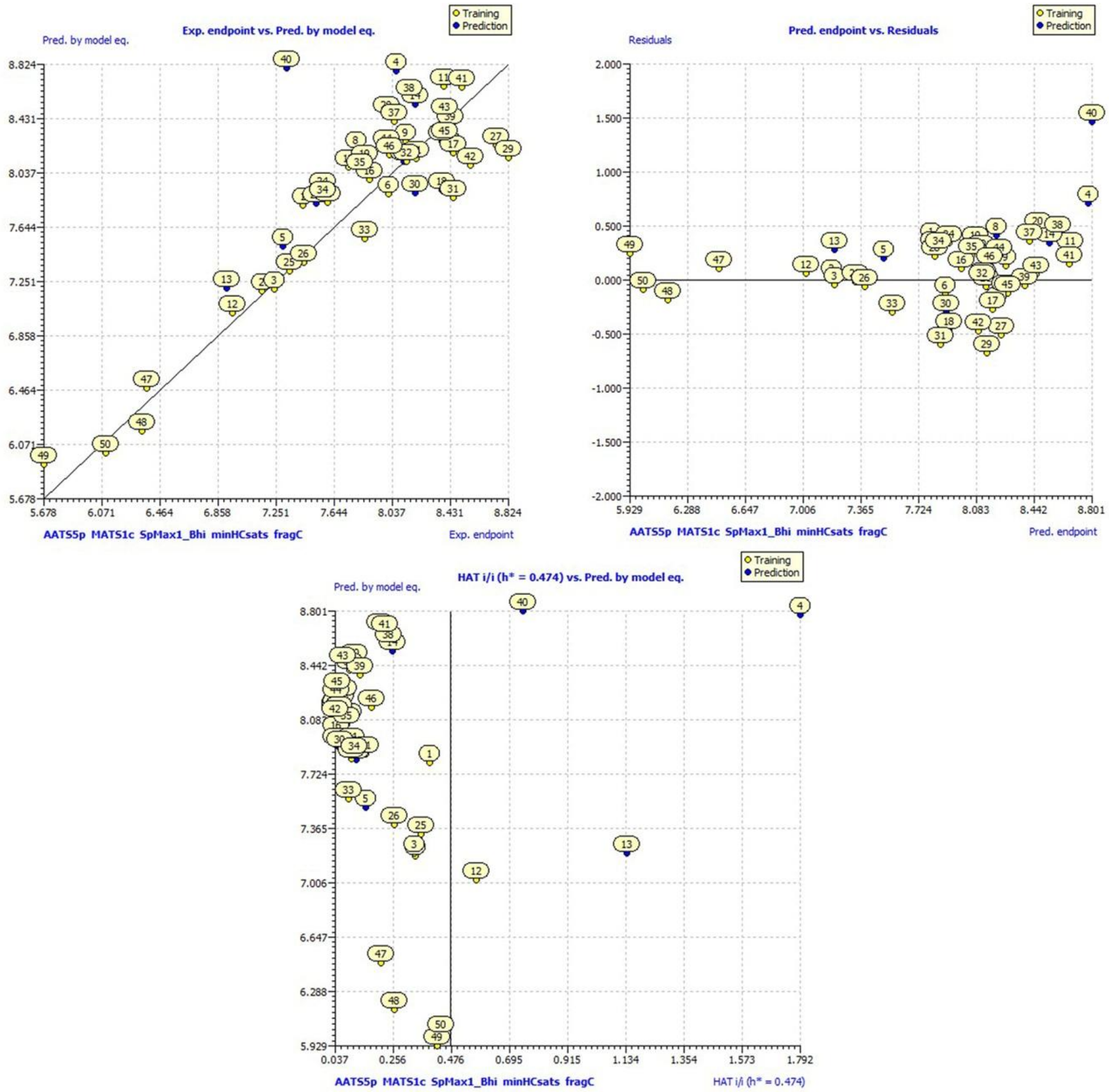

Figure S3. Above left) Graphical representation of developed QSAR model with GA-MLR method for split 3; Above right) Difference between experimental and calculated pIC50 values; Bellow) Graphical representation of applicability domain established for split 3.

Table S3. The list of SAks together with their correlation weights for the three runs of the Monte Carlo optimization

SAk	CW(SAk)			SAk	CW(SAk)			SAk	CW(SAk)		
	Run 1	Run 2	Run 3		Run 1	Run 2	Run 3		Run 1	Run 2	Run 3
#...........	0.56022	-0.18436	-0.76744	P4E0C...3-..	0.44833	0.58891	0.29669	PT3-N...5...	2.14626	1.09536	2.41613
11,000,000	0.04525	-0.36638	0.16871	P4E0C...4-..	-0.72547	-0.99392	-0.99433	PT3-N...6...	2.29091	0.06492	0.68924
11,001,000	-0.90278	0.2839	0.1755	P4E0C...5-..	2.33747	-0.62316	1.22869	PT3-N...7...	0.46526	-0.22928	0.48622
11,001,100	2.0345	1.60809	-0.85535	P4E0C...6-..	-0.82147	-0.70026	-0.19798	PT3-O...2...	-0.36204	0.49353	-0.33019
1,011,001,000	0.57547	1.35149	-0.71975	P4E0C...7-..	-0.96797	-0.89119	0.22039	PT3-O...3...	-0.36486	0.09418	0.94066
1,011,001,100	3.25145	2.31921	0.65163	P4E0C...8-..	1.51631	0.09422	0.23873	PT3-O...4...	2.26555	-0.40233	1.23286
10,011,001,000	-0.65856	2.04741	0.17824	P4E0C...9-..	-1.10661	-0.7471	-0.77826	PT3-O...5...	6.58776	1.20189	6.07775
(...(.......	-0.56149	0.95031	-0.52897	P4E0F...1-..	3.33372	2.55391	4.483	PT3-o...4...	2.15169	-0.53975	-0.60697
(...........	0.22588	0.4677	0.25374	P4E0F...3-..	-0.4859	1.31873	0.18514	PT3-s...4...	2.4973	2.41927	-0.53176
(...C...#...	-0.42973	-0.00262	-0.67879	P4E0F...4-..	2.35194	2.39086	0.29951	PT4-C...10..	2.03789	2.34105	0.02624
(...C...(...	0.05324	-0.81419	0.46211	P4E0F...7-..	-0.80866	0.21133	1.49781	PT4-C...11..	-0.25932	-0.65256	-1.37064
(...F...(...	-0.96151	-0.88159	-0.60856	P4E0Cl..1-..	0.35249	2.53709	3.91191	PT4-C...12..	-0.66508	0.04676	0.45609
(...N...#...	-1.0356	-0.56972	-1.01918	P4E0Cl..4-..	-0.82012	-1.091	0.07806	PT4-C...13..	0.6505	-0.12867	-0.68281
(...c...(...	0.46392	-0.6127	0.05553	P4E0N...0-..	-0.57704	2.20395	0.42657	PT4-C...14..	-0.40166	-0.72759	-0.52546
++++F---B2==	2.0823	-0.76391	0.46506	P4E0N...1-..	-0.41223	0.13723	0.86739	PT4-C...15..	-0.51853	-0.23154	-0.90337
++++F---B3==	0.49408	0.63931	0.40086	P4E0N...10-.	-1.49215	-0.58928	0.12084	PT4-C...16..	-0.73023	-0.26676	0.0455
++++F---Cl==	2.29661	-0.23547	-0.81074	P4E0N...12-.	1.22475	-0.03805	0.3288	PT4-C...2...	0.25421	2.29743	0.26786
++++F---N===	-0.93254	-0.51516	-0.5737	P4E0N...2-..	0.50425	0.2633	2.41365	PT4-C...3...	-0.87692	-1.01107	-0.22566
++++F---O===	3.37238	-0.0834	2.14279	P4E0N...3-..	2.13375	0.63417	0.29633	PT4-C...4...	1.11162	-0.62642	-1.10296
++++F---S===	0.73714	-0.46022	1.69102	P4E0N...4-..	-1.2158	1.01993	-0.54153	PT4-C...5...	-0.07991	2.31157	2.46664
++++CL--N===	2.03894	2.22164	-0.52204	P4E0N...6-..	-0.96553	0.16181	0.1635	PT4-C...6...	0.07146	-0.39924	-0.71048
++++CL--O===	2.056	0.49952	0.44833	P4E0N...7-..	1.03473	-0.63443	2.34372	PT4-C...7...	2.23434	-1.69766	0.08065
++++Cl--B3==	1.51188	0.0332	2.03982	P4E0O...0-..	1.35447	2.37544	0.49281	PT4-C...8...	-1.0995	-2.64828	-2.97485
++++N---B2==	2.04456	-0.59419	0.45121	P4E0O...1-..	-1.49681	-2.5026	-0.69128	PT4-C...9...	0.48494	0.31304	0.00328
++++N---B3==	2.19939	0.49703	-0.85055	P4E0O...11-.	8.73838	5.90668	7.13832	PT4-F...2...	2.4962	4.47701	1.3534
++++N---O===	0.05494	2.10483	0.75413	P4E0O...2-..	-0.61598	1.64978	-0.65457	PT4-F...4...	-0.48396	0.32166	0.06658
++++N---S===	2.17034	1.50537	-0.03175	P4E0O...3-..	-0.44717	0.83836	1.01704	PT4-F...5...	0.04851	2.46111	2.01536

++++O---B2==	1.11138	1.04276	1.3451	P4E0O...4-..	0.70838	-0.85317	-0.76765	PT4-F...8...	-0.68497	-0.19409	0.36614
++++O---B3==	-0.8624	-0.91933	-0.9218	P4E0o...4-..	-1.06844	-0.56199	1.38794	PT4-Cl..2...	1.89293	1.43722	0.01818
++++O---S===	0.26227	2.02725	2.02272	P4E0s...4-..	2.46429	1.88098	2.49294	PT4-Cl..5...	-1.94173	1.9022	0.36198
1...(...(...	0.40381	2.02608	0.672	NNC-C...101.	2.46439	-0.69692	1.21122	PT4-N...13..	0.51292	0.54586	0.32677
1...(.......	2.03665	2.40907	-0.82416	NNC-C...110.	0.04815	-0.88621	-0.97103	PT4-N...14..	-0.28976	1.13458	-0.50487
1...........	-0.83976	-0.91378	-0.61299	NNC-C...202.	0.47767	2.29488	0.64364	PT4-N...2...	-4.04717	-4.24934	-0.68225
1...C...(...	1.69162	-0.92619	4.74145	NNC-C...211.	-1.02013	1.13649	0.18858	PT4-N...4...	-0.85216	-0.55051	-0.22138
1...F...(...	1.20943	-1.24084	2.26346	NNC-C...220.	0.01062	-0.58043	0.46344	PT4-N...5...	2.5299	-0.61187	2.48064
1...Cl..(...	3.65676	3.2144	-1.423	NNC-C...312.	-0.1239	0.37429	-0.53024	PT4-N...6...	2.36147	0.49542	-0.45478
1...c...(...	-1.02679	-0.73877	-0.64554	NNC-C...321.	-0.59331	-1.34085	0.48686	PT4-N...8...	0.17493	0.49478	-0.80506
1...c...1...	2.01799	0.03332	-0.88484	NNC-C...330.	0.42525	-1.00891	-0.99956	PT4-N...9...	0.41818	0.35927	0.46779
1...n...(...	2.3303	0.37374	2.1578	NNC-C...413.	-0.50086	0.24967	-0.82189	PT4-O...13..	3.03354	5.28988	4.06384
2...(...(...	-0.54343	-1.02552	0.32154	NNC-C...440.	1.5395	1.1107	0.98808	PT4-O...2...	-0.90807	-0.24943	-0.88104
2...(.......	0.10467	2.03318	0.12309	NNC-F...110.	1.32737	0.18961	-0.7981	PT4-O...4...	2.2989	0.0317	-1.33331
2...........	0.27378	0.31642	2.38407	NNC-Cl..110.	1.24543	0.10947	0.23068	PT4-O...5...	2.20147	-0.92357	-0.04266
2...c...(...	2.39375	-0.86769	-0.68577	NNC-N...110.	-0.91828	0.25902	2.08277	PT4-O...6...	-0.62156	0.29559	2.21663
=...(.......	-0.67378	-0.46813	0.26211	NNC-N...202.	0.37001	-0.83405	0.05411	PT4-o...6...	2.19454	2.1441	-0.5971
=...........	0.2254	-0.04606	0.17804	NNC-N...211.	0.10962	2.37759	0.03601	PT4-s...6...	-0.51568	2.17117	0.7367
=...N...(...	2.31587	2.25188	-0.56831	NNC-N...220.	0.66352	1.47207	0.08515	Nmax.1......	1.36941	-0.33983	0.16821
=...O...(...	0.52448	2.07277	0.07624	NNC-N...312.	2.34172	1.24697	0.4525	Nmax.2......	1.82454	1.21266	-0.59603
C...#.......	0.37297	2.02662	0.50289	NNC-N...321.	-0.82549	-0.80171	0.20656	Nmax.5......	0.1059	2.42869	-0.12155
C...(...(...	2.08396	1.10712	2.44039	NNC-N...330.	0.21154	-0.63393	-0.75217	Omax.1......	1.25439	-1.2602	0.56657
C...(.......	-0.7175	0.0016	0.2494	NNC-O...101.	1.31207	0.35747	-0.66193	Omax.2......	0.56457	-0.59627	-0.64387
C...(...1...	2.32186	0.24619	1.71394	NNC-O...110.	-0.98299	-0.94777	-0.91233	Omax.3......	-0.92559	0.36153	0.6247
C...(...2...	1.78506	2.42704	-1.0679	NNC-O...220.	0.30717	0.15855	0.34137	VS2-C...10..	0.11644	0.27772	0.33323
C...(...=...	0.2147	-1.59524	-0.83684	NNC-o...220.	1.26882	-0.75188	0.58642	VS2-C...11..	0.07955	-0.3773	0.10197
C...(...C...	0.3386	-0.50435	0.11274	NNC-s...220.	3.32085	2.24767	0.2062	VS2-C...12..	1.49673	0.61878	-0.93467
C...........	-0.62617	-0.92754	-0.79326	NNE0C...100-	2.01122	2.21615	1.45554	VS2-C...3...	-0.41039	-0.60557	1.45749
C...1...(...	2.19043	-0.96583	-0.56915	NNE0C...109-	-0.89222	-0.94217	-0.8138	VS2-C...4...	-0.0788	-0.52693	1.60697
C...1.......	-0.59061	0.20627	2.30968	NNE0C...200-	2.33712	0.67753	0.33218	VS2-C...5...	-0.54203	-0.15928	-0.04786
C...2...(...	0.47837	0.426	-0.7744	NNE0C...209-	0.43085	-1.19714	-1.11525	VS2-C...6...	2.32342	0.03549	2.4139

C...2.......	0.08509	0.47826	2.16138	NNE0C...218-	0.11477	2.01121	0.40049	VS2-C...7...	2.20916	0.76448	0.14239
C...C...(...	1.22926	0.1352	0.24291	NNE0C...309-	1.16057	0.03522	0.00496	VS2-C...8...	-0.74446	0.10116	-0.33595
C...C.......	-0.86323	0.18346	-0.3647	NNE0C...318-	-0.54027	0.08641	-0.84484	VS2-C...9...	-1.26722	-0.69513	-0.51307
C...C...1...	-0.59116	-0.22451	-0.61296	NNE0C...327-	0.08976	-0.86299	0.3803	VS2-F...4...	2.9285	3.0584	3.68858
C...C...2...	-0.5924	0.48846	2.29375	NNE0C...409-	-0.04679	0.10196	-0.64865	VS2-F...5...	0.30313	-0.91834	-0.5171
C...C...C...	-0.73677	-1.01265	-0.83054	NNE0C...436-	6.29833	1.90959	0.50714	VS2-F...6...	-0.57862	0.27946	-0.62335
C...N...(...	-0.0385	0.12192	-0.71795	NNE0F...109-	0.46906	0.4321	1.03008	VS2-Cl..4...	1.13348	0.84293	1.84595
C...N...1...	0.69876	-2.05797	-1.14381	NNE0Cl..109-	2.328	-0.28268	2.01838	VS2-Cl..5...	-1.47557	-0.67081	0.44086
C...N...C...	2.44998	1.70923	0.30546	NNE0N...109-	-0.8281	-0.45255	-0.02978	VS2-N...12..	2.11174	-0.01946	2.35046
C...O...(...	0.26488	2.51108	-0.58807	NNE0N...200-	-1.12419	0.2116	0.05066	VS2-N...3...	-0.78551	0.04281	0.45209
C...O...2...	2.67906	0.75655	0.02001	NNE0N...209-	-0.75997	2.13327	-0.5558	VS2-N...4...	2.45229	0.51573	-0.11907
C...O...C...	-1.92646	-0.26022	0.15273	NNE0N...218-	1.78285	1.05703	-0.17517	VS2-N...5...	2.36237	-0.60848	0.45258
C...c...1...	1.31266	1.11408	0.68948	NNE0N...309-	2.00041	0.33049	0.24042	VS2-N...6...	-0.94162	0.18881	-0.0099
C...n...1...	-0.93201	0.04846	0.22064	NNE0N...318-	-0.357	-0.71597	-0.96585	VS2-N...7...	0.0721	-0.6975	-0.10407
C3......0...	0.36363	2.46596	2.08586	NNE0N...327-	0.23322	-0.21394	1.40054	VS2-N...8...	-0.47582	-0.70179	-0.65576
C4......0...	2.13902	2.92208	6.28373	NNE0O...100-	1.21009	-0.2868	0.50153	VS2-N...9...	1.73293	-0.95619	-0.71542
C5......0...	1.8329	0.33721	2.06484	NNE0O...109-	-0.99756	-0.83608	-0.94869	VS2-O...4...	-1.07222	0.57763	0.32034
C5....H.1...	2.25616	2.02521	1.18885	NNE0O...218-	0.11267	-0.77293	0.41771	VS2-O...5...	-1.40322	-4.06165	2.21931
C5...AH.1...	0.20762	-0.63671	-0.57625	NNE0o...218-	-0.12253	0.02724	0.2899	VS2-O...7...	-0.88789	1.37374	-0.56438
C6...AH.3...	0.45407	0.35006	2.02489	NNE0s...218-	2.24796	2.00906	3.21694	VS2-o...7...	-1.56468	0.63843	1.30894
C6...AH.4...	5.0157	1.2952	2.84498	NOSP11000000	6.33652	5.86711	2.8346	VS2-s...7...	2.1711	1.0133	0.35845
C7......0...	6.23735	6.25774	4.35824	S2E0C...0-..	-0.34334	0.37335	-0.84575	VS3-C...10..	0.09213	0.12056	1.04484
BOND00000000	-0.93571	2.09108	0.13549	S2E0C...1-..	-0.74704	1.33898	-0.31825	VS3-C...11..	-0.77487	0.40612	-0.43551
BOND01000000	-0.95509	-0.81457	0.20156	S2E0C...2-..	1.03635	0.36314	2.27686	VS3-C...12..	0.00796	-0.93669	-0.43899
BOND10000000	0.67914	-0.8428	1.39522	S2E0C...3-..	0.23933	0.33236	0.1129	VS3-C...13..	-0.82829	-0.71341	-0.99246
F...(...(...	0.07765	-0.64575	-0.8407	S2E0C...4-..	2.3657	1.01254	0.93092	VS3-C...14..	1.17101	-0.9228	-0.0402
F...(.......	0.9043	0.26783	0.25846	S2E0C...5-..	0.50098	0.70592	1.21359	VS3-C...15..	-0.91716	-1.26526	-0.69312
F...(...C...	-0.04606	2.09196	0.96204	S2E0C...6-..	-0.53036	0.09386	-0.41292	VS3-C...16..	-0.27043	0.27843	2.5885
F...(...F...	-0.57205	-1.17457	-0.63208	S2E0C...7-..	-0.45481	-1.5069	-0.05453	VS3-C...17..	-0.6108	-0.69708	0.07095
F...........	0.23244	0.35374	0.59169	S2E0C...8-..	-0.90197	-1.337	-0.56268	VS3-C...18..	-0.62523	-0.628	0.32764
F...1.......	-0.68984	-0.82889	0.35469	S2E0C...9-..	-0.80131	1.5199	-0.26889	VS3-C...4...	-0.67112	1.36376	-0.6998

F...C...(...	-0.53978	-0.41681	1.34304	S2E0F...3-..	3.33172	3.04561	0.77712	VS3-C...5...	3.09695	2.43528	0.3268
F...C.......	0.05546	-0.90832	-0.85385	S2E0F...4-..	-0.64693	0.05064	-0.85958	VS3-C...6...	2.34564	2.12325	2.49833
F...c...1...	2.69579	0.51834	1.93475	S2E0F...5-..	0.12892	1.06164	-0.83194	VS3-C...7...	2.36119	2.01904	-0.47534
EC0-C...1...	0.08597	0.26595	0.41304	S2E0Cl..3-..	1.60612	2.81217	1.48865	VS3-C...8...	0.51665	-0.4504	0.17133
EC0-C...2...	2.44342	-0.83953	-0.58674	S2E0Cl..4-..	2.12891	1.7508	2.13689	VS3-C...9...	2.25613	0.26936	-0.14933
EC0-C...3...	-0.94265	-1.45472	-0.75561	S2E0N...1-..	3.15499	3.01527	4.27254	VS3-F...10..	-0.61836	0.18502	-0.78175
EC0-C...4...	-0.84257	0.01002	0.43087	S2E0N...2-..	1.00622	0.45511	0.26148	VS3-F...4...	3.36209	1.74123	2.40492
EC0-F...1...	-0.75032	0.17272	0.13381	S2E0N...3-..	0.93391	-1.03789	-0.0473	VS3-F...5...	1.41036	0.40908	-0.80393
EC0-Cl..1...	1.91953	0.64135	2.38262	S2E0N...4-..	0.13644	-0.65594	-0.76802	VS3-F...7...	2.1577	2.09062	2.48696
EC0-N...1...	0.16273	0.28788	0.45481	S2E0N...5-..	-0.89835	-1.95944	-0.05271	VS3-F...8...	0.41929	2.25932	0.26519
EC0-N...2...	0.27493	0.25583	0.28907	S2E0N...6-..	1.46232	1.10017	1.11726	VS3-F...9...	-0.80776	0.03668	0.28775
EC0-N...3...	-0.66052	-0.63771	-0.50944	S2E0N...7-..	0.01862	-0.94812	-0.55152	VS3-Cl..4...	0.64693	3.31202	-0.07066
EC0-O...1...	-1.28822	-0.65291	-0.87497	S2E0N...9-..	-0.16418	-0.2112	-0.58824	VS3-Cl..7...	0.42397	2.40505	-0.81416
EC0-O...2...	0.39446	0.25953	-0.81703	S2E0O...2-..	-0.87825	-0.43866	-0.5886	VS3-N...10..	-0.69953	2.97575	-0.74165
EC0-o...2...	0.76125	0.44076	-0.92708	S2E0O...3-..	-0.6444	2.25981	1.60274	VS3-N...11..	-0.5476	2.00314	-0.50492
EC0-s...2...	1.51481	1.83009	-0.16401	S2E0O...4-..	-1.99043	-0.99416	1.07547	VS3-N...13..	0.36161	-0.31874	0.14815
Cl..(.......	0.3192	0.09329	1.7152	S2E0O...5-..	-0.57968	0.37095	2.03858	VS3-N...4...	0.91532	-2.09656	-0.43407
Cl.........	1.86783	2.12188	-0.34062	S2E0o...5-..	0.51146	2.33441	-1.03659	VS3-N...5...	2.08294	-0.42772	0.87175
Cl..1.......	1.83177	-0.82614	0.51478	S2E0s...5-..	2.12708	2.69841	0.23703	VS3-N...6...	-0.91753	-0.83128	-0.63462
Cl..c...1...	0.89558	0.96046	2.31683	S3E0C...0-..	0.00864	-0.56612	-0.89867	VS3-N...7...	2.49144	1.96019	0.61369
Cmax.1......	-0.52989	0.27353	-0.07716	S3E0C...1-..	-0.99274	-0.70043	-0.54266	VS3-N...8...	-0.30515	1.21237	0.06325
Cmax.2......	1.04357	2.60515	2.21789	S3E0C...10-.	-0.62643	-0.98869	0.25802	VS3-N...9...	1.23637	2.35031	2.03823
HALO00000000	1.90614	-0.23937	2.39382	S3E0C...11-.	-0.50617	-0.79687	-0.22993	VS3-O...10..	0.08867	0.09596	-0.75818
HALO10000000	0.26399	-0.62055	-1.0944	S3E0C...12-.	-1.37217	-1.78532	-0.20206	VS3-O...11..	0.10517	-1.01006	0.07283
HALO11000000	-0.80603	2.36802	2.60651	S3E0C...13-.	5.38156	0.91359	2.26825	VS3-O...12..	2.08354	0.25805	0.41153
N...#.......	0.26723	2.21717	-0.81948	S3E0C...14-.	-0.94444	1.10228	0.15459	VS3-O...4...	-0.93413	1.998	-0.79862
N...#...C...	-0.6498	2.32251	1.08969	S3E0C...15-.	0.49774	0.22148	0.57013	VS3-O...5...	1.62685	2.9261	1.17227
N...(.......	1.27721	1.21366	1.80609	S3E0C...2-..	-0.17246	2.42691	0.317	VS3-O...6...	-0.27053	1.23061	-0.94513
N...(...=...	2.49588	1.86324	0.84111	S3E0C...3-..	0.29599	0.24263	-0.04111	VS3-O...9...	0.04539	-0.69279	-0.65974
N...(...C...	-0.6065	-0.27177	-0.75271	S3E0C...4-..	1.08182	-0.21011	0.38978	VS3-o...8...	0.14295	1.31477	-0.33733
N...........	0.299	0.14015	2.41368	S3E0C...5-..	0.30457	1.37466	-0.07059	VS3-s...8...	2.62276	-0.02243	0.58076

N...1...(...	1.25273	1.9698	-0.37901	S3E0C...6-..	2.3467	-0.57626	1.92242	Smax.0......	3.08664	4.22935	2.76068
N...1.......	-0.41876	-0.57995	-0.68004	S3E0C...7-..	2.02073	0.33981	1.01821	c...(.......	0.35139	-0.11446	-0.80818
N...1...C...	0.09162	-0.62289	0.90619	S3E0C...8-..	0.48069	0.40395	0.00209	c...(...1...	-0.36625	1.36188	2.37815
N...=.......	2.05084	-0.713	0.14	S3E0C...9-..	-0.46339	0.53375	-0.3277	c...(...2...	2.4969	0.31541	0.41643
N...C.......	0.22561	0.73706	-0.57069	S3E0F...3-..	0.76612	2.46665	0.51986	c...(...C...	2.61166	-0.66228	-0.97479
N...C...C...	2.33487	1.33266	1.97416	S3E0F...4-..	0.31734	0.13493	-0.716	c...(...F...	0.1418	-0.97044	-0.76158
N...N.......	2.03327	2.27463	-0.45064	S3E0F...6-..	1.98712	2.16353	2.42779	c...(...Cl..	2.99502	2.33827	1.50876
N...N...1...	1.16699	2.29574	-0.05005	S3E0F...7-..	-0.61926	2.61068	-0.60073	c...(...N...	-0.78781	0.03273	0.24869
N...N...N...	2.27126	0.13344	-0.4879	S3E0F...8-..	-0.45407	-0.87196	1.33009	c...(...O...	0.19047	0.70613	-0.54927
O...(.......	-0.94872	-0.66915	1.0737	S3E0F...9-..	0.12032	-0.92071	-0.84246	c...(...c...	0.43075	0.4388	0.14804
O...(...1...	-0.33118	-0.54722	-0.50946	S3E0Cl..3-..	2.09239	1.73646	2.93223	c..........	0.05877	0.44037	0.03012
O..........	-0.50864	-0.91835	-0.78849	S3E0Cl..6-..	2.0283	-0.82968	0.33662	c...1...(...	-0.50617	-0.79696	1.17655
O...2...(...	2.24101	-0.9382	-0.81582	S3E0N...1-..	2.43553	1.8932	0.21094	c...1.......	-0.70324	-0.59183	0.05318
O...2.......	1.47484	0.69091	-0.19661	S3E0N...10-.	-0.74726	-0.58156	-0.57567	c...1...C...	-0.28253	3.33284	1.43113
O...=...(...	1.2641	-0.66935	0.40876	S3E0N...11-.	-0.89699	0.08611	0.20081	c...1...F...	0.23269	2.00472	-0.56217
O...=.......	0.41174	2.43173	-0.75926	S3E0N...2-..	-0.50816	3.25696	1.33511	c...1...Cl..	-0.80503	-0.72116	-0.97989
O...=...N...	2.05187	2.24879	1.10945	S3E0N...3-..	2.40161	-0.52331	1.31323	c...1...c...	0.14102	0.26163	-0.7459
O...C...(...	-6.1881	-4.87994	-2.11838	S3E0N...4-..	2.10029	0.42101	2.04308	c...2...(...	0.31389	0.14319	-0.95039
O...C.......	2.08576	1.11644	0.498	S3E0N...5-..	0.25503	0.19703	2.12494	c...2.......	-0.38713	0.26475	-0.79418
O...C...1...	2.39324	-1.29079	-0.4204	S3E0N...6-..	2.16331	2.16481	0.6584	c...2...c...	2.98691	-0.2937	0.25524
O...C...C...	2.46955	2.36773	-0.57653	S3E0N...8-..	0.26575	2.35828	-0.77691	c...C...#...	0.32647	2.46339	0.32685
O...C...O...	-0.53506	0.08361	-0.71303	S3E0N...9-..	-0.5383	-0.89282	-1.04517	c...C.......	0.6344	-0.8426	2.91382
O...c...1...	-0.10496	-1.09437	-0.74964	S3E0O...10-.	-0.79832	-0.69671	2.46499	c...C...O...	-0.97358	-0.83509	0.32126
P2E0C...0-..	0.72141	1.96163	0.03309	S3E0O...2-..	-0.76842	-3.33578	-0.09186	c...F.......	2.23435	0.03555	1.89135
P2E0C...1-..	-0.91979	0.06641	-0.92475	S3E0O...3-..	-2.8697	-1.04668	1.91519	c...Cl......	3.06857	-1.15893	-0.79291
P2E0C...2-..	-3.05547	-1.21713	-0.92625	S3E0O...4-..	3.12867	4.08749	0.27312	c...O.......	0.01878	-1.0922	-0.59799
P2E0C...3-..	-0.99073	-0.99579	-0.7464	S3E0O...7-..	-0.3991	-0.3937	-0.5117	c...O...C...	0.16991	1.50435	0.23471
P2E0F...1-..	2.18722	0.71877	0.24291	S3E0O...8-..	-0.20918	-0.08778	0.32379	c...c...(...	1.40678	-0.85784	0.21933
P2E0F...2-..	-0.0932	0.25369	0.30463	S3E0O...9-..	2.39444	-0.70413	0.27028	c...c.......	-0.99537	0.18408	0.32734
P2E0Cl..1-..	0.04676	2.2622	-0.48922	S3E0o...6-..	2.23259	1.2824	1.01437	c...c...1...	2.35402	0.23013	0.46321
P2E0N...0-..	-0.07398	2.10756	-0.03796	S3E0s...6-..	2.64892	2.59152	2.64955	c...c...2...	-0.11866	0.36267	-0.02442

P2E0N...1-..	1.26825	0.07321	-0.19851	PT2-C...1...	1.26551	-0.82181	-0.96526	c...c...c...	-0.73709	2.35029	-0.5679
P2E0N...2-..	-0.78971	-0.58015	0.5882	PT2-C...2...	-0.6867	0.49369	0.42404	c...n...(...	1.86061	2.05329	0.30213
P2E0O...0-..	1.6736	-0.65684	1.9606	PT2-C...3...	1.38248	0.16015	0.20237	c...n...1...	0.24882	0.62358	-0.28716
P2E0O...1-..	-0.99608	-0.86673	0.35027	PT2-C...4...	-0.0921	0.04528	0.09038	c...n...c...	-4.43438	-0.95103	0.18932
P2E0o...1-..	1.15894	-0.85419	-0.92589	PT2-C...5...	-1.49524	-3.40338	1.55216	c...o...1...	2.08554	0.45968	0.72843
P2E0s...1-..	3.4635	3.06183	1.5562	PT2-C...6...	1.352	-0.03741	0.49875	c...s...1...	-0.41267	2.26139	1.3785
P3E0C...0-..	-0.27609	0.2095	-0.95911	PT2-F...2...	-0.53947	2.0078	1.25626	n...(.......	2.05153	0.28607	0.33472
P3E0C...1-..	-0.26879	-0.58719	0.42409	PT2-F...3...	-1.01238	-0.84305	-0.68204	n...(...1...	0.02559	0.29544	2.18571
P3E0C...2-..	1.44183	1.9857	0.24558	PT2-Cl..2...	2.49446	0.3466	-0.1223	n...(...c...	2.54477	2.08748	0.15722
P3E0C...3-..	0.65263	2.37821	2.14446	PT2-N...1...	0.29496	-0.80546	-0.41442	n...........	0.32236	-0.10262	0.36188
P3E0C...4-..	-0.68755	-1.01454	0.00336	PT2-N...2...	2.69203	0.92251	2.4364	n...1...(...	2.25945	-2.93785	0.40727
P3E0C...5-..	4.24737	6.34478	2.67801	PT2-N...3...	-0.05468	-0.0627	0.17566	n...1.......	1.3839	0.32213	0.10461
P3E0F...1-..	2.33263	0.09482	0.38364	PT2-N...4...	-0.53238	-1.29009	-2.55711	n...1...c...	1.36067	0.08624	0.49381
P3E0F...2-..	1.00955	-0.01895	-0.29489	PT2-N...5...	0.62594	-0.97489	0.97752	n...1...n...	-0.56068	0.36963	0.16998
P3E0F...3-..	1.18968	-0.81301	-0.75845	PT2-O...2...	2.25436	-0.83197	0.37899	n...C.......	0.16698	-1.10598	1.39942
P3E0Cl..1-..	3.37481	1.68786	2.42563	PT2-O...3...	-0.46498	0.56495	2.62776	n...c...(...	-0.53057	1.67729	0.59315
P3E0Cl..2-..	-0.81235	-0.71462	-0.70283	PT2-o...3...	0.55572	0.61934	2.21138	n...c.......	0.48968	0.2049	0.46536
P3E0N...0-..	2.27341	2.17403	2.36857	PT2-s...3...	2.02722	0.52509	2.39424	n...c...1...	-0.0656	-0.97736	0.32313
P3E0N...1-..	-0.51142	-0.4511	0.33543	PT3-C...2...	1.96086	0.4166	0.09723	n...c...c...	-0.15571	-0.36671	0.37888
P3E0N...2-..	0.26345	0.41091	0.19978	PT3-C...3...	2.21408	0.44195	-0.0394	n...n.......	0.03024	-0.79632	-0.42704
P3E0N...3-..	0.21964	1.09288	-0.56675	PT3-C...4...	2.1838	2.37628	2.70521	n...n...1...	-0.55998	0.72045	-0.72816
P3E0N...4-..	-0.9682	2.35312	-0.57373	PT3-C...5...	-0.51544	2.16215	0.17127	n...n...c...	-1.04528	0.21793	0.29966
P3E0O...0-..	-1.03423	-1.83537	0.06795	PT3-C...6...	-0.88296	-0.73143	0.13713	n...n...n...	0.8372	-0.47139	0.47213
P3E0O...1-..	2.31132	-1.2034	0.59611	PT3-C...7...	0.10672	0.44201	-0.8915	o...........	2.18585	2.70678	2.47876
P3E0O...2-..	0.25377	0.17341	0.48049	PT3-C...8...	-0.98775	-0.88357	0.18081	o...1...(...	1.82184	1.24044	0.93891
P3E0O...3-..	3.45009	2.71489	3.90632	PT3-F...2...	0.32314	0.77623	0.20667	o...1.......	2.35101	-0.24358	0.17552
P3E0o...2-..	1.80665	0.57034	-0.40366	PT3-F...3...	2.39641	2.4777	0.0108	o...c.......	-0.31264	2.52089	0.70035
P3E0s...2-..	2.00547	1.8482	2.37638	PT3-F...4...	0.11454	-0.21928	0.12334	o...c...c...	2.35103	0.49628	-0.65498
P4E0C...0-..	0.48175	1.24679	3.85332	PT3-Cl..2...	-0.85648	0.57314	0.05954	s...........	-0.17783	-1.0856	2.60503
P4E0C...1-..	2.1255	-0.02856	0.71194	PT3-Cl..3...	2.77871	1.62074	3.80839	s...1...(...	1.66044	1.9667	0.50738
P4E0C...10-.	0.17489	-0.44577	0.72739	PT3-N...2...	2.16572	0.37171	0.00823	s...1.......	2.14295	-0.43538	2.12671

P4E0C...12-.	-0.94841	-0.6297	-0.00102	PT3-N...3...	2.33969	1.13764	2.04717	s...c......	3.57915	2.37687	0.13422
P4E0C...13-.	5.54585	5.44216	1.67194	PT3-N...4...	-0.46782	-1.00886	-0.98895	s...c...c...	2.69814	0.62186	0.70279
P4E0C...2-..	-0.78836	0.02849	1.12765								

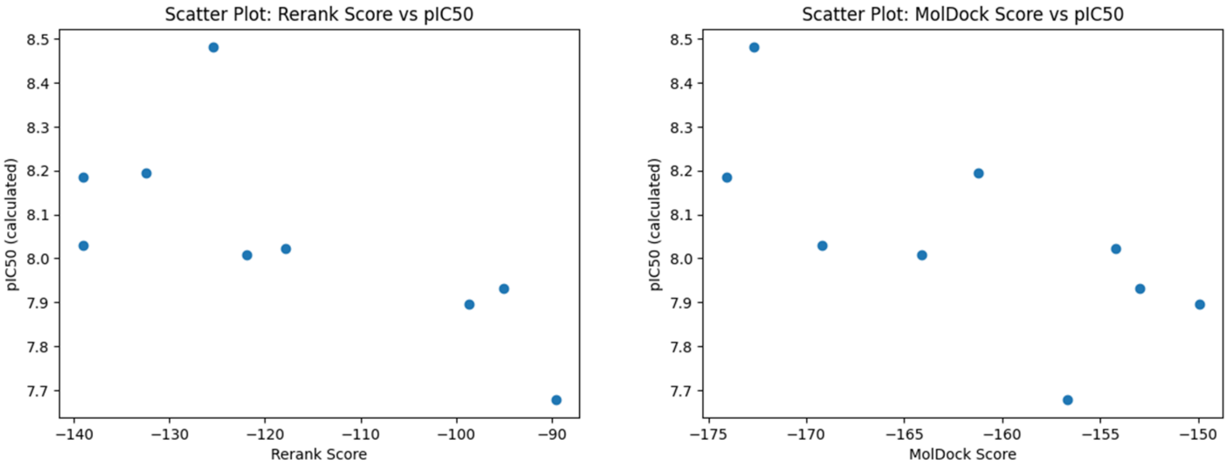

Figure S4. Correlation analysis between docking scores and calculated pIC50 values for compounds A–A8.

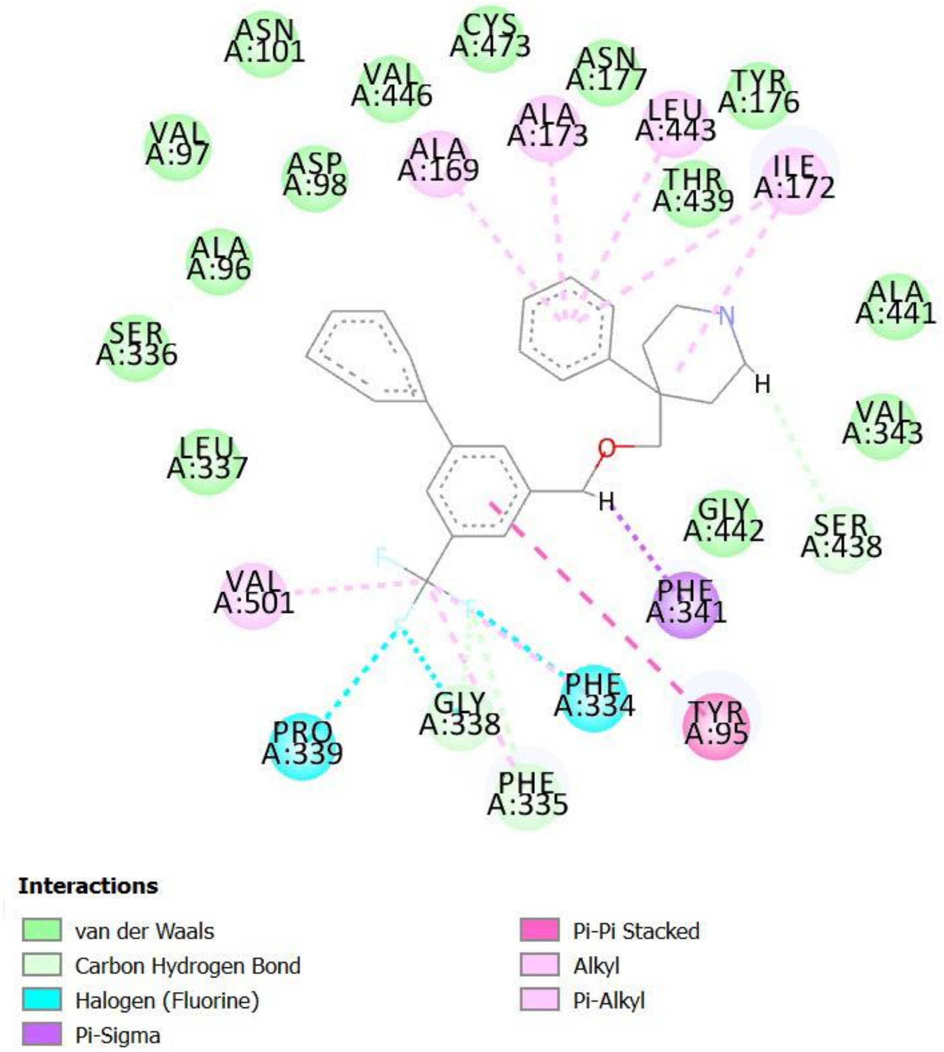

Figure S5. Two-dimensional representation of the interaction between molecule A and amino acids inside serotonin transporter binding pocket.

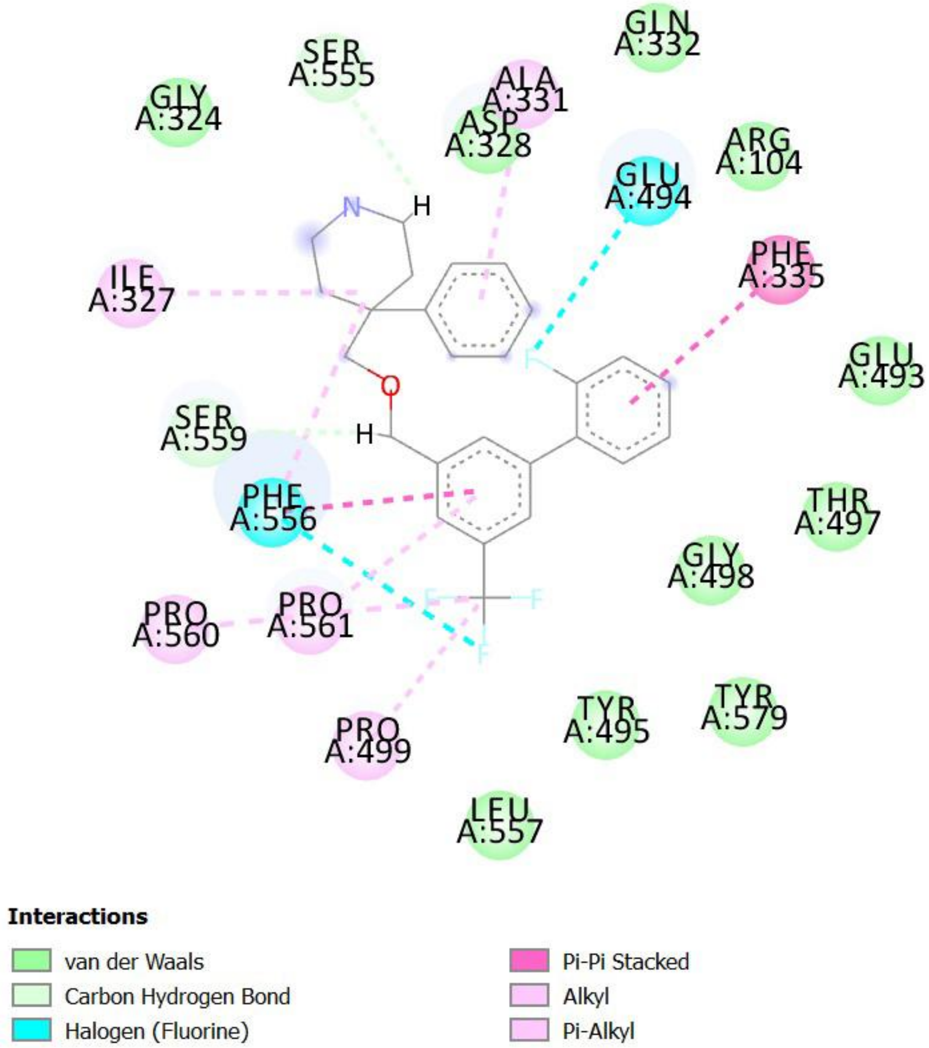

Figure S6. Two-dimensional representation of the interaction between molecule A1 and amino acids inside serotonin transporter binding pocket.

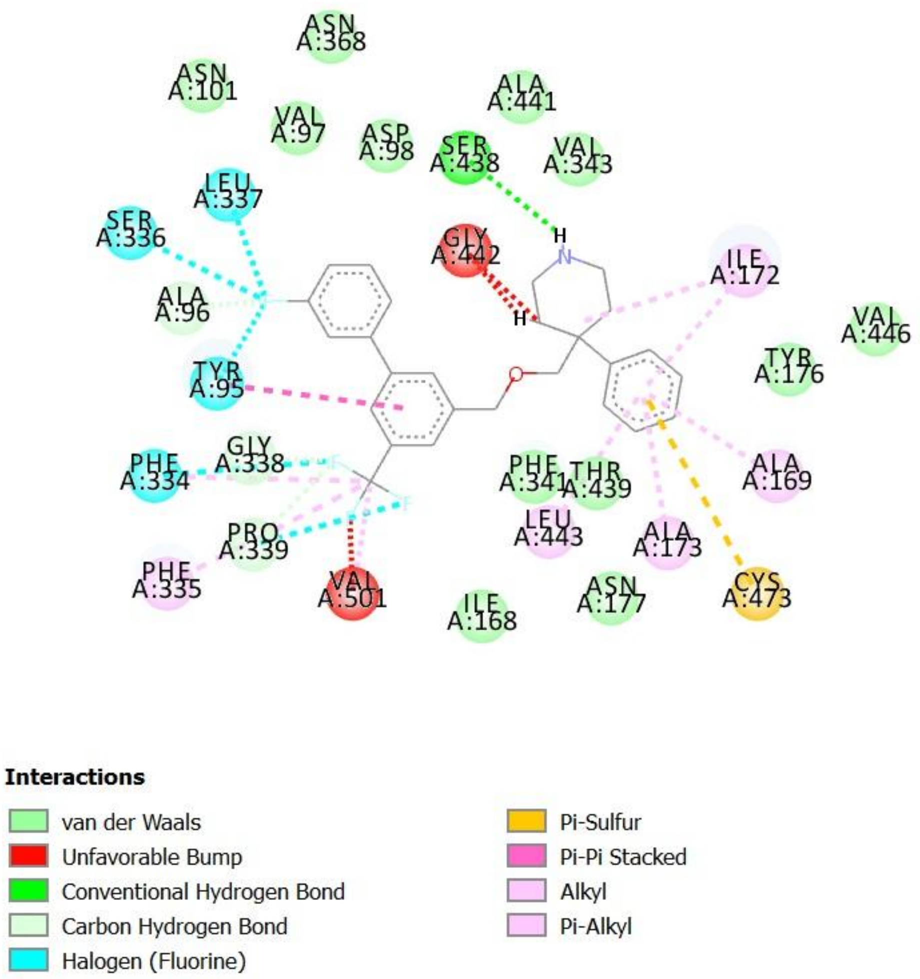

Figure S7. Two-dimensional representation of the interaction between molecule A2 and amino acids inside serotonin transporter binding pocket.

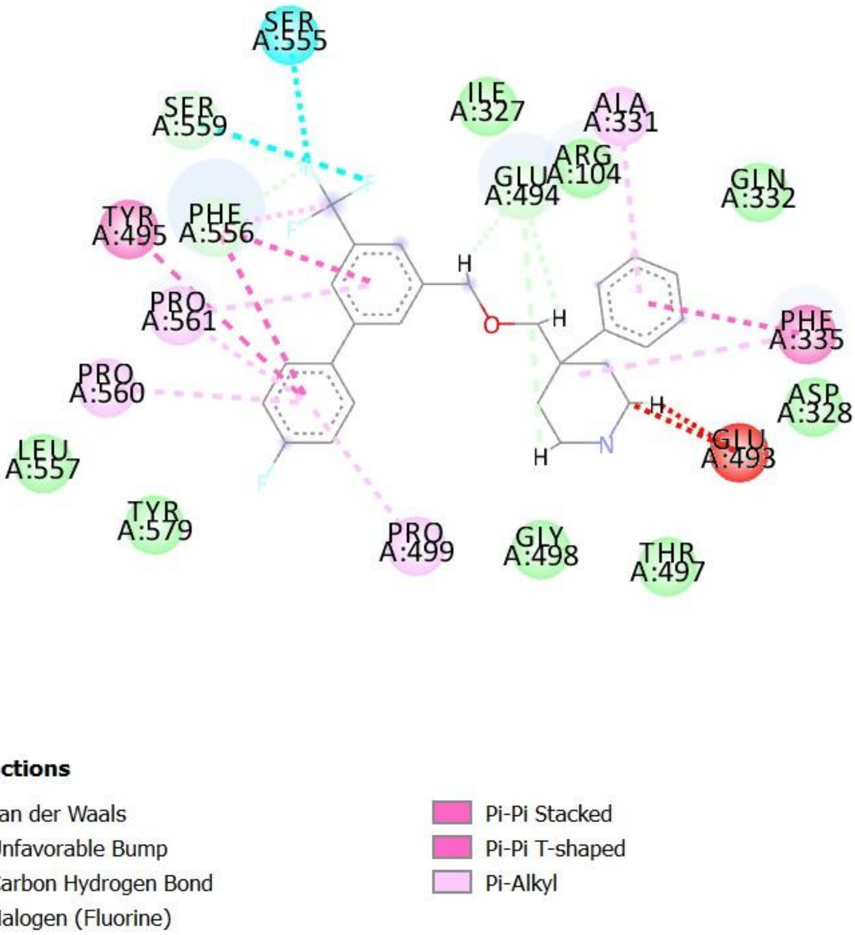

Figure S8. Two-dimensional representation of the interaction between molecule A3 and amino acids inside serotonin transporter binding pocket.

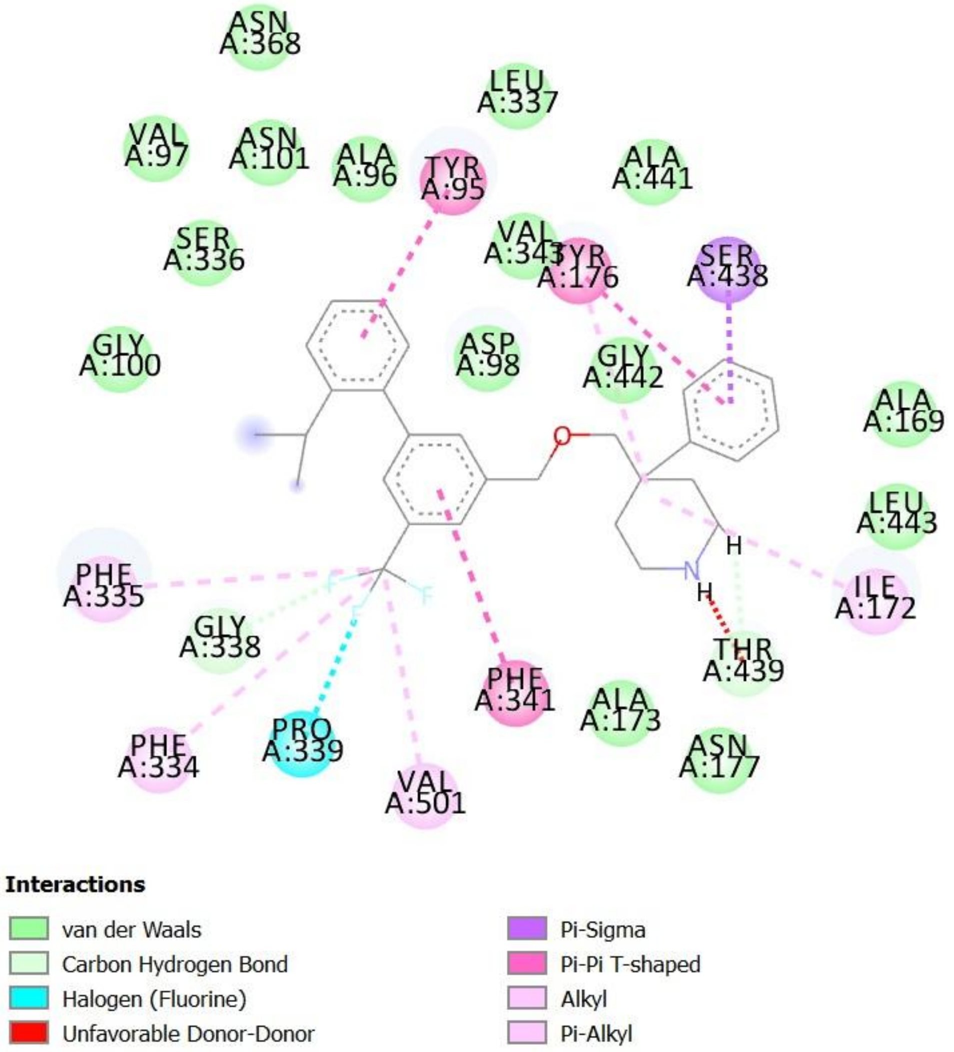

Figure S9. Two-dimensional representation of the interaction between molecule A4 and amino acids inside serotonin transporter binding pocket.

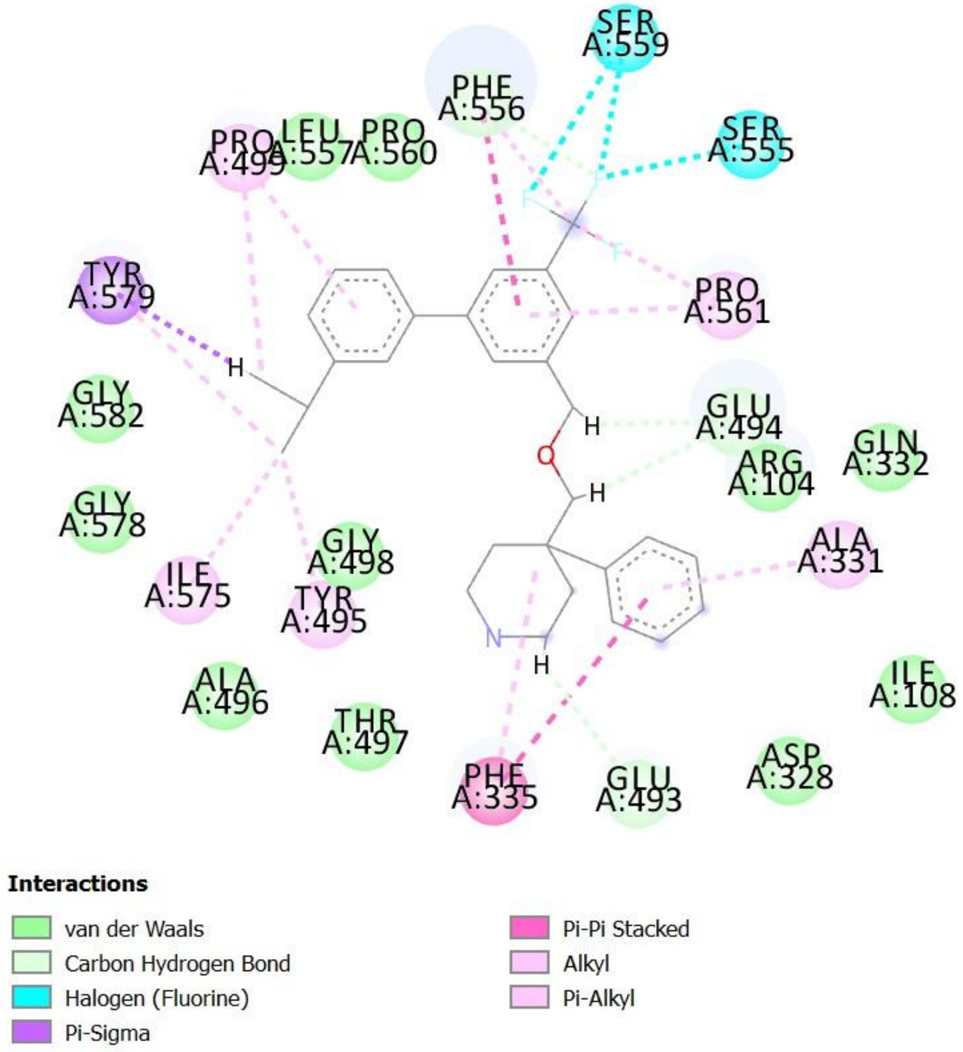

Figure S10. Two-dimensional representation of the interaction between molecule A5 and amino acids inside serotonin transporter binding pocket.

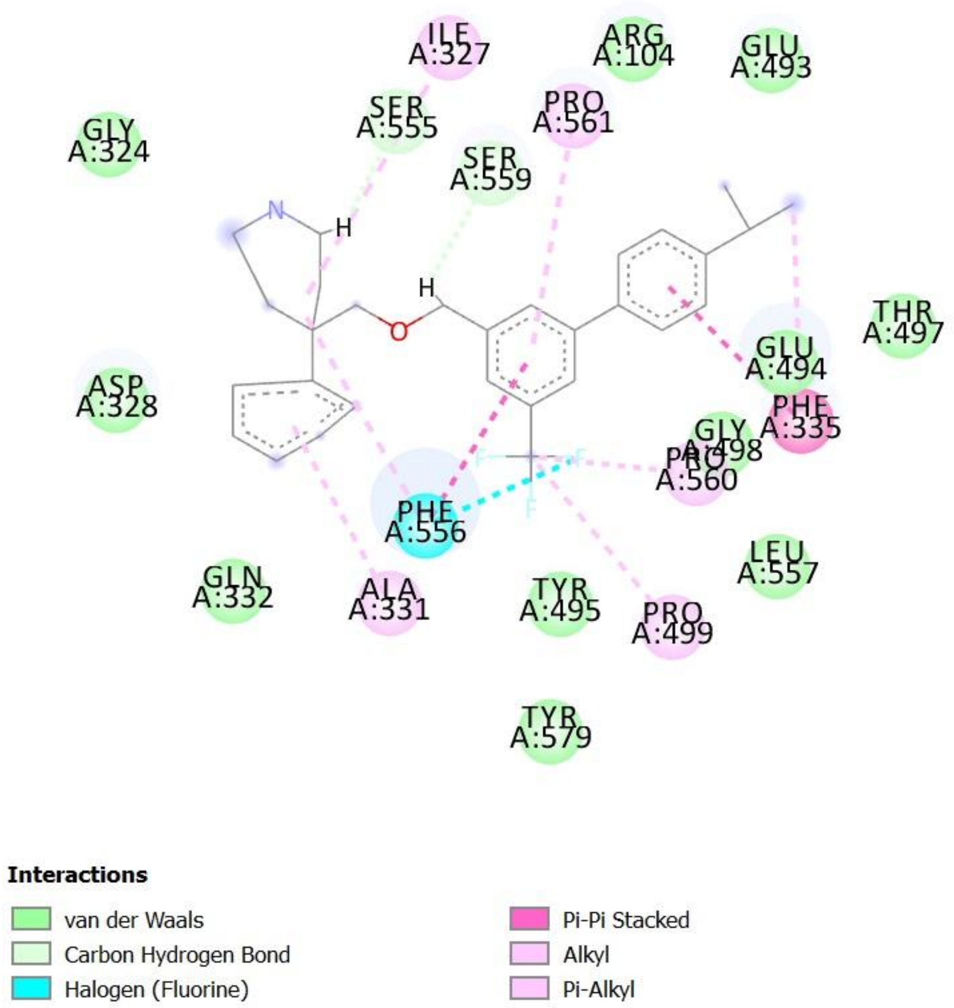

Figure S11. Two-dimensional representation of the interaction between molecule A6 and amino acids inside serotonin transporter binding pocket.

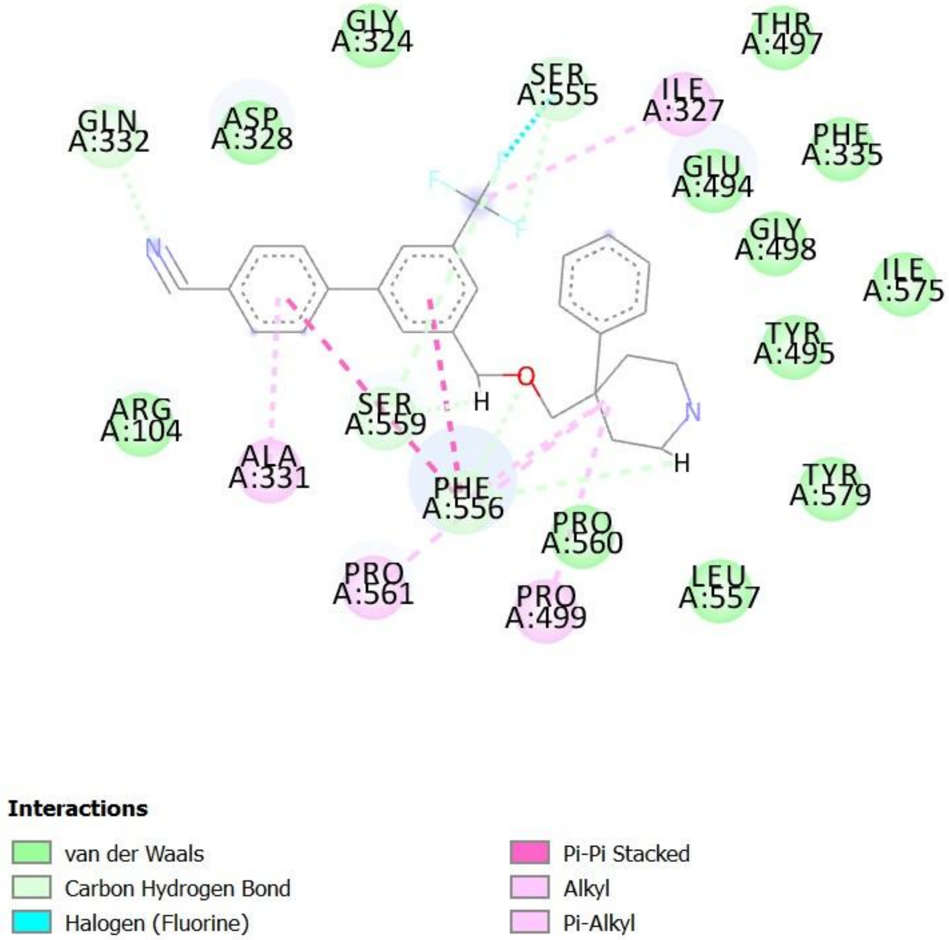

Figure S12. Two-dimensional representation of the interaction between molecule A7 and amino acids inside serotonin transporter binding pocket.

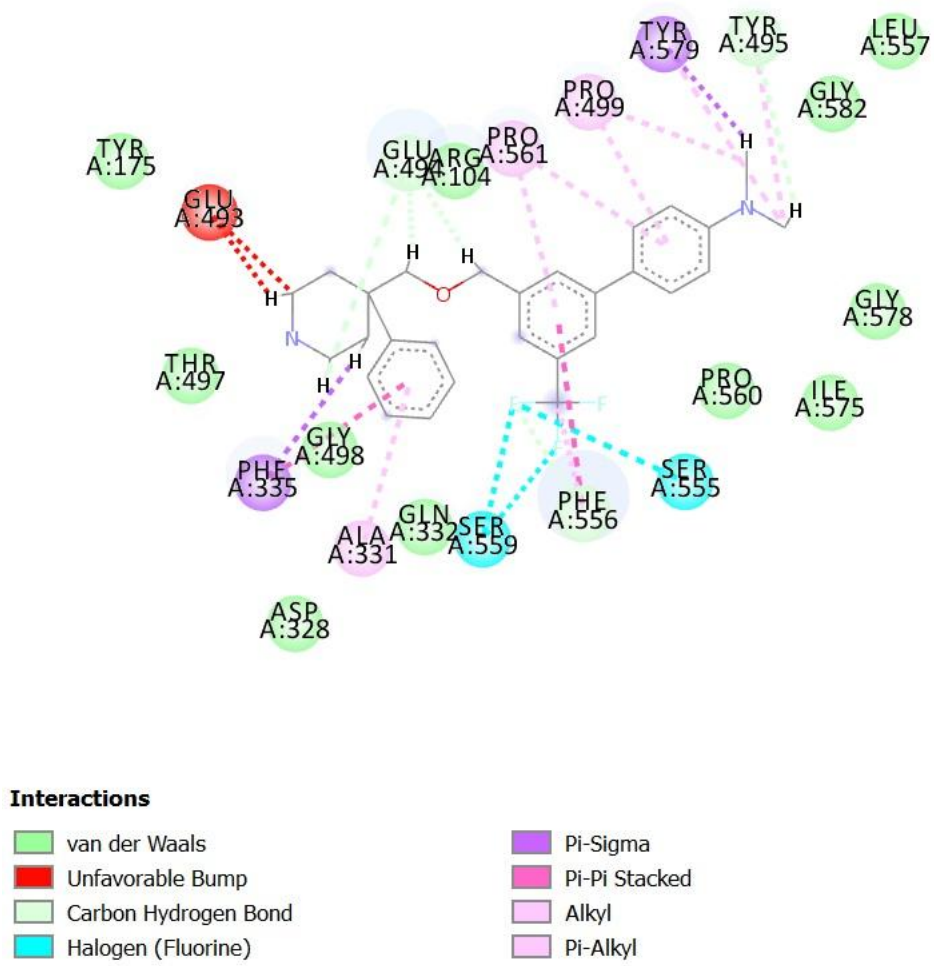

Figure S13. Two-dimensional representation of the interaction between molecule A8 and amino acids inside serotonin transporter binding pocket.

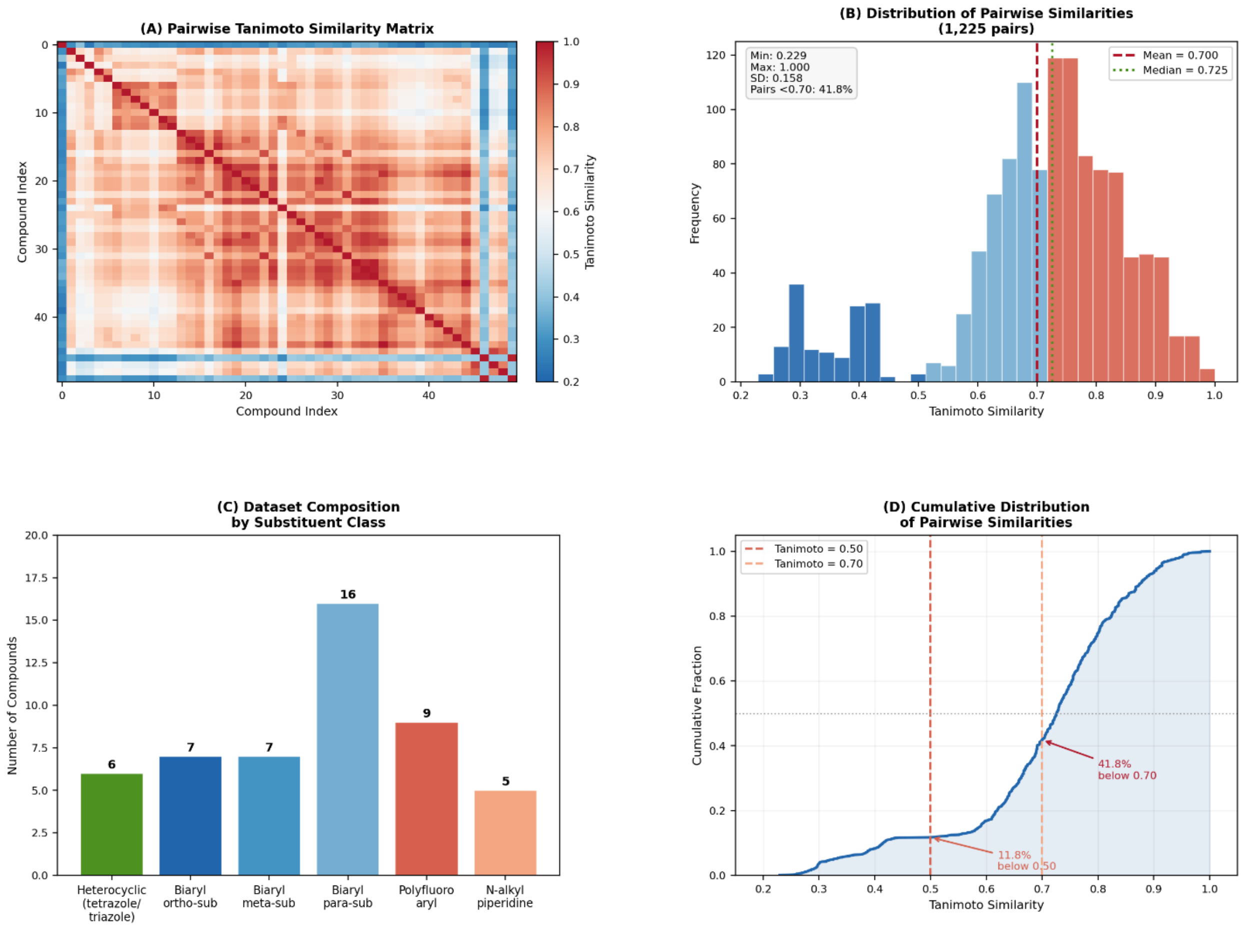

Figure S14. Structural diversity analysis of the QSAR dataset (n = 50 compounds). (A) Pairwise Tanimoto similarity matrix computed from SMILES-based structural fingerprints for all 50 compounds (1,225 unique pairs). Color scale ranges from blue (low similarity) to red (high similarity). (B) Frequency distribution of pairwise Tanimoto similarity values. Mean similarity = 0.700 (SD = 0.158); 41.8% of compound pairs exhibit Tanimoto similarity below 0.70, indicating meaningful structural variation within the dataset. (C) Composition of the dataset by substituent class, illustrating the systematic exploration of six distinct structural subgroups. (D) Cumulative distribution of pairwise similarities; 11.8% of pairs fall below Tanimoto = 0.50, confirming the presence of structurally distinct compound clusters, particularly among heterocyclic derivatives.
